# X-ray-driven nanomotor with enhanced penetration and retention for carbon monoxide-amplified radioimmunotherapy of advanced colorectal cancer

**DOI:** 10.1016/j.mtbio.2025.102398

**Published:** 2025-10-13

**Authors:** Xin Zhao, Huayi Sun, Zikun Shen, Shaowen Wang, Fangman Chen, Xiaochun Xie, Shuhui Wang, Yucen Zhang, Yan Guo, Yidan Zhang, Quanxin Ning, Dan Shao, Hong Zhang

**Affiliations:** a Department of General Surgery, Shengjing Hospital of China Medical University, Shenyang, 110004, China; b National Engineering Research Center for Tissue Restoration and Reconstruction, South China University of Technology, Guangzhou, 510006, China; c Department of Anesthesiology, The Fifth Clinical Medical College of Inner Mongolia Medical University, Hohhot, 010110, China

**Keywords:** Nanomotor, Gas therapy, Radiosensitization, Immunotherapy, Advanced colorectal cancer

## Abstract

Most advanced colorectal cancer (CRC) with peritoneal metastasis have been managed by radiotherapy and following localized perfusion of therapeutic agents. However, the efficacy of intraperitoneal treatment remains limited by poor drug penetration, inadequate retention within tumor tissues, and a complex tumor microenvironment. Here we report the development of an X-ray-activated nanomotor, GM-R848, comprising an iron carbonyl (FeCO) prodrug framework encapsulating the TLR7 agonist resiquimod (R848), designed for efficient tumor cell elimination and immune activation. Upon X-ray irradiation, rapid decomposition of the FeCO framework generates carbon monoxide (CO) bubbles, propelling enhanced penetration and retention of the nanomotors within colorectal tumor tissues. Following internalization, CO amplifies DNA damage to sensitize tumor cells to radiotherapy, thereby inducing immunogenic cell death. Concurrently, the sequential release of R848 stimulates robust immune activation, synergistically enhancing anti-tumor immunity within the peritoneal cavity. This integrated radio-gas-immunotherapy strategy achieved a 95.3 % tumor growth inhibition rate in an advanced CRC model while mitigating adverse effects associated with radiotherapy and immunotherapy. These findings create a framework for X-ray-driven nanorobots in precision oncology, offering a promising approach for the targeted management of advanced cancers.

## Graphical abstract

**Figure:**
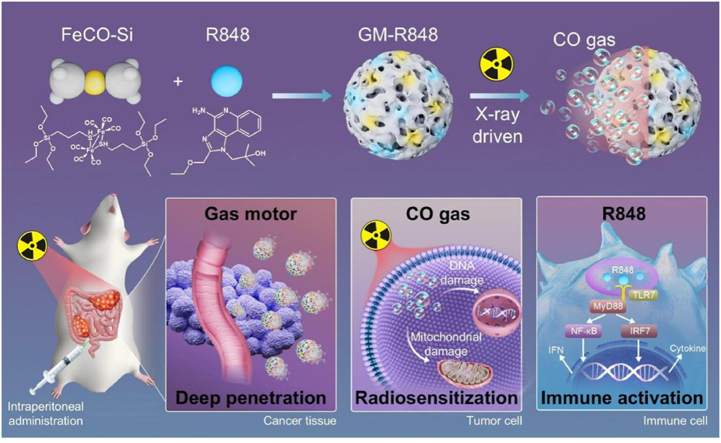

## 1 Introduction

Colorectal cancer (CRC) persists as a leading malignancy worldwide, with persistently high prevalence, and mortality rates despite significant progress in diagnosis and treatment [1,2]. The progression of peritoneal metastases in advanced CRC substantially worsens patient prognosis, and the 5-year survival rate is below 10 %, driven by challenges in surgical resectability and the limitations of current treatments, including localized chemotherapy, immunotherapy, and radiotherapy [3,4]. The efficacy of these interventions is hindered by inadequate drug targeting and retention within tumor tissues, rapid clearance from the peritoneal cavity, and a complex tumor microenvironment (TME) that poses significant barriers to effective localized drug delivery [[5], [6], [7]].

To overcome these limitations, nanoparticle-based strategies, including pH-sensitive, redox-responsive, or receptor-mediated targeting systems, which improve drug accumulation in tumor tissues [[8], [9], [10], [11]]. However, most nanomedicines rely on passive diffusion, which is insufficient in overcoming rapid clearance and the intricate TME of the peritoneal cavity, resulting in suboptimal retention and barrier penetration [[12], [13], [14], [15]]. Consequently, there is an urgent need for innovative delivery platforms to improve the penetration and retention of therapeutic agents in cancer cells.

Self-propelling micro/nanomotors (MNMs) have emerged as promising drug delivery vehicles due to their capabilities in directed navigation, enhanced biological barrier penetration, and efficient cargo transport [[16], [17], [18], [19], [20], [21]]. Gas-driven MNMs, powered by biocompatible gases such as H_2_, CO, or NO, can be activated by internal stimuli (e.g., pH, redox, or enzymatic triggers), enabling on-site propulsion with high biocompatibility [[22], [23], [24], [25], [26]]. Additionally, external stimuli, including light, electricity, and ultrasound, can remotely trigger gas release, bypassing TME dependency [[27], [28], [29], [30], [31], [32], [33]]. However, reports on X-ray-driven self-propelling micro/nanomotors, particularly for localized radioimmunotherapy of advanced CRC, remain scarce [[34], [35], [36]].

**Scheme 1:**
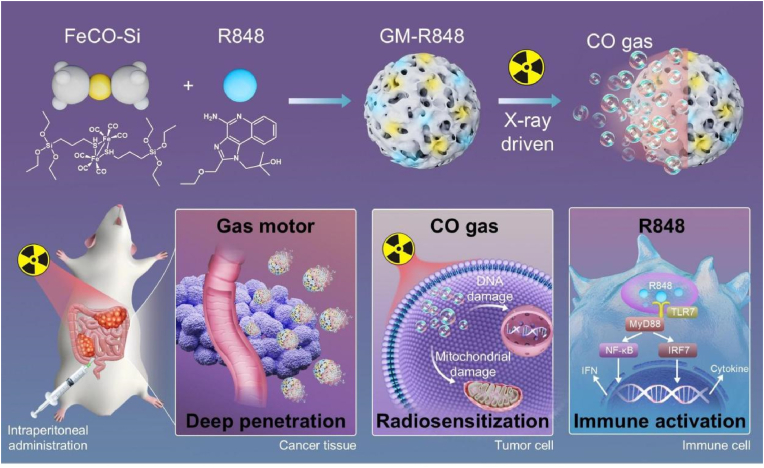
Schematic illustration of the GM-R848 synthesis and the anti-tumor strategy. Upon X-ray irradiation, GM-R848 rapidly generates CO bubbles, potentiating enhanced penetration and retention within colorectal tumor tissues. CO potentiates radiosensitization by amplifying DNA damage, while concurrently released R848 activates robust anti-tumor immunity, synergistically suppressing advanced CRC progression.

Here, we present an X-ray-activated nanomotor designed for targeted and effective radioimmunotherapy of advanced CRC. As illustrated in Scheme 1, these nanomotors, formed via self-assembly of iron carbonyl (FeCO)-bridged silane monomers and the TLR7 agonist resiquimod (R848), enable precise targeting of cancer cells under X-ray irradiation, facilitating efficient cargo delivery, tumor cell elimination, and immune activation. The FeCO-bridged framework rapidly generates CO bubbles upon X-ray exposure, driving enhanced penetration and retention within colorectal tumor tissues. Following cellular uptake, sustained CO release amplifies DNA damage, sensitizing tumor cells to radiotherapy and promoting immunogenic cell death (ICD). Concurrently, the sequential release of R848 stimulates robust immune activation, synergistically eliciting an anti-tumor immune response and optimizing the peritoneal TME. This integrated radio-gas-immunotherapy approach significantly suppresses advanced CRC progression and metastasis while minimizing systemic toxicity. The X-ray-driven nanomotors achieve codelivery of gas and immunomodulatory agents, combining enhanced tumor penetration, synergistic radio-responsive gas therapy, and potent immunotherapy. These findings provide crucial insights into the application of X-ray-driven gas-propelling nanomotors and highlight their potential for clinical translation in the management of advanced CRC.

## 2 Experimental section

### 2.1 Synthesis and characterization of GM and GM-R848

FeCO-bridged organosilane precursor (FeCO-Si) was synthesized according to a previously established method [37]. GM-R848 was synthesized via a modified sol-gel approach. Cetyltrimethylammonium bromide (CTAB, 0.6 g) and triethanolamine (TEAH, 0.2 g) were dissolved in deionized water (40 mL) with stirring at 800 rpm. The solution was heated in a thermostated oil bath at 80 °C for 30 min. Subsequently, a silica precursor mixture of tetraethyl orthosilicate (TEOS), R848, and FeCO-Si (4 g total mass) was added dropwise (1 mL/min) via syringe pump. The reaction was maintained at 80 °C for 4 h with continuous stirring. The resulting GM-R848 nanomotors were harvested at 10000×*g* for 5 min and purified through three ethanol washes (40 mL each). UV–vis spectroscopy quantified the R848 absorbance against a standard curve. The loading efficiency (LE) and loading content (LC) of R848 were determined by measuring the unbound R848 in the combined supernatants and wash solutions, applying the established R848 calibration. LE and LC were calculated according to the following equations: LE (%) = (total R848 - free R848)/total R848 × 100 %, LC (%) = (total R848 - free R848)/weight of GM-R848 × 100 %.

The GM-R848 were then dispersed in anhydrous ethanol and refluxed under condenser for 48 h to eliminate surfactants. SEM, TEM, FTIR, and UV–vis spectroscopy were used to characterize the GM-R848 structure. ICP-MS measurements of iron content determined the CO loading efficiency by quantifying the Fe_3_(CO)_12_ incorporated into GM-R848.

The GM nanoparticles were synthesized using an identical protocol to that of GM-R848, with the sole exception that R848 was omitted from the silica precursor mixture.

### 2.2 The X-ray-triggered release and degradation of GM-R848

A standard curve for CO was constructed based on the fluorescent intensity (at 513.5 nm) of FL-CO-1. To evaluate the effect of X-rays on CO release, a GM-R848 solution (1 mg/mL) was irradiated with 2 Gy of X-rays. Subsequently, the irradiated solution was treated with FL-CO-1 (10 μmol/L) and PdCl_2_ (10 μmol/L). At predetermined time points, the fluorescent intensity at 513.5 nm was measured to calculate the amount of CO released by reference to the standard curve. TEM imaging further characterized the structural changes in GM-R848 during degradation.

### 2.3 Synthesis of GM-R848-FITC and GM-R848-ICG

To synthesize APTES-FITC/APTES-ICG, fluorescein isothiocyanate (FITC, 4 mg) or indocyanine green (ICG, 4 mg) and 3-aminopropyltriethoxysilane (APTES, 200 μL) were dissolved in anhydrous dimethyl sulfoxide (DMSO, 2 mL). The mixture was then reacted for 2 h under nitrogen. For GM-R848-FITC/GM-R848-ICG synthesis, we slowly added the APTES-FITC/APTES-ICG solution to the mixed silica precursors (TEOS, R848, and FeCO-Si) to form a homogeneous mixture. The remaining steps followed the same procedure as GM-R848 synthesis. We collected the final product by centrifugation and stored it at 4 °C.

### 2.4 Stability of GM-R848 *in vitro*

To check the time-course stability of GM-R848 in different media, GM-R848 was dispersed in PBS with or without 10 % FBS. At specific time intervals, GM-R848 were isolated through centrifugation (10000 rpm, 5 min). The diameter and zeta potential of GM-R848 were measured by dynamic light scattering (DLS) to evaluate the stability *in vitro*.

### 2.5 UHPLC analysis

A 3.5 μm Waters XBridge™ C18 column (2.1 × 150 mm) separated and quantified R848 at a flow rate of 1 mL/min with detection at 227 nm. Mobile phase A contained 10 mM sodium phosphate with 0.1 % triethylamine, adjusted to pH 2.45, while mobile phase B was pure acetonitrile. The 6.5-min gradient program maintained 15 % mobile phase B from 0 to 3 min, increased to 45 % from 3 to 5 min, and returned to 15 % from 5.1 to 6.5 min. After initial dilution in acetonitrile, samples were further diluted in 30 % acetonitrile and 70 % 0.1 N hydrochloric acid before injection, following calibration with six R848 standards prepared in pure acetonitrile.

### 2.6 Pharmacokinetics

Mice received intraperitoneal injections of either free R848 (10 mg/kg) or GM-R848 (800 mg/kg), with or without X-ray irradiation (2 Gy). Blood samples were collected via cardiac puncture at 10 min, 1, 2, 6, and 12 h post-intraperitoneal injection, then centrifuged at 4 °C to isolate plasma. For peritoneal fluid collection, 2 mL PBS was injected into the peritoneal cavity and gently massaged to facilitate fluid expansion. R848 concentrations in plasma and peritoneal fluid were quantified using UHPLC.

### 2.7 TLR7 agonist activity assay

The nanomotors' TLR7 agonist activity was evaluated in HEK-Blue hTLR7 reporter cells. HEK-Blue hTLR7 reporter cells were treated with various formulations containing equivalent R848 concentrations for 24 h. Following the manufacturer's protocol, we quantified SEAP reporter activity employing 620 nm wavelength absorption of culture supernatants.

### 2.8 Immunogenic cell death (ICD) effect *in vitro*

CT26 cells were cultured in plates and treated with PBS, GM or GM-R848 (50 μg/mL). The X-ray irradiation group then received 2 Gy irradiation, followed by 24 h of incubation. For calreticulin (CRT) detection, cells were stained with an anti-CRT antibody for 30 min prior to flow cytometric analysis. HMGB1 secretion was quantified by measuring supernatant concentrations using an ELISA kit with microplate reader detection.

### 2.9 *In vitro* differentiation of BMDCs and BMDMs from bone marrow mononuclear cells

Following CO_2_ euthanasia, femurs and tibiae were harvested from C57BL/6 mice for bone marrow extraction by repeated flushing with PBS using syringes. The cells treated with sterile red blood cell lysis buffer were then cultured in complete RPMI-1640 medium containing 20 ng/mL GM-CSF and 10 ng/mL IL-4. For dendritic cell differentiation (BMDCs), cells were cultured with 20 ng/mL GM-CSF and 10 ng/mL IL-4; for macrophage differentiation (BMDMs), 20 ng/mL M-CSF was substituted. On day 2, 50 % of the medium was replaced with fresh cytokine-supplemented medium, followed by full medium replacement on day 4. After 8 days of culture, BMDCs (non-adherent/weakly adherent) were collected by gentle pipetting, while adherent BMDMs were detached using cell scrapers. All cells were centrifuged for downstream applications.

### 2.10 IFN-γ ELISpot assay

The Multiscreen HTS-IP plate was activated with 70 % ethanol, rinsed with DPBS, and coated overnight at 4 °C with anti-mouse IFN-γ capture antibody. After blocking with sterile 10 % FBS in RPMI-1640 for 2 h, spleens from OT-I mice were harvested, mechanically dissociated, and processed to yield single-cell suspensions. Sterile RBC lysis buffer lysed RBC, and splenocytes were cultured in RPMI-1640 full medium. BMDCs, pre-stimulated for 24 h with supernatants from CT26-OVA cells (ovalbumin-transfected CT26), were added at 2 × 10^5^ cells/well. Positive control wells received splenocytes stimulated directly with anti-mouse CD3*ε* (145-2C11) and anti-mouse CD28 (37.51) antibodies (1:1000). Following 48 h incubation, media were removed, and plates underwent sequential incubation with biotinylated anti-IFN-γ detection antibody, streptavidin-HRP conjugate, and AEC substrate. After air-drying, spots were quantified using a CTL ImmunoSpot® S6 Analyzer.

### 2.11 *In vitro* assessment of chemodynamic therapy (CDT) and radiosensitization effects

RAW 264.7 murine macrophages and HIEC-6 human intestinal epithelial cells were plated in complete media and cultured for 24 h treated with GM-R848 or GM + R848 (0–800 μg/mL) at equivalent R848 concentrations. For cytotoxicity comparison, CT26 cells were treated with GM-R848 (0–800 μg/mL), while the X-ray irradiation group received 2 Gy radiation. Cell viability was assessed via MTT assay, with absorbance measurements taken at 490 nm and 570 nm.

### 2.12 Intracellular reactive oxygen species (ROS) radical and CO detection

CT26 cells were cultured with GM-R848 or GM (50 μg/mL) with or without X-ray irradiation (2 Gy) followed by 24 h incubation. To remove extracellular nanoparticles, cells underwent three sequential washes with PBS. Cells were then treated with the fluorescent probe 2′,7′-dichlorofluorescin diacetate (DCFH-DA, 10 μM), which oxidizes to green-fluorescent DCF upon ROS interaction. The production of CO was measured using a fluorogenic CO probe (FL-CO-1). Fluorescence images were acquired by CLSM and analyzed using ImageJ.

### 2.13 DNA damage analysis

CT26 cells were processed using the same protocol as for intracellular ROS radical detection. Cells were fixed in 4 % paraformaldehyde (30 min) and permeabilized with PBS containing 1 % Triton X-100 (1 h). The cells were then stained overnight at 4 °C with 500 μL anti-γH_2_AX antibody (1:500 dilution). After PBS washing, the cells were incubated with EGFP-conjugated sheep anti-rabbit secondary antibody (1:1000 dilution) at 37 °C for 1 h. Excess antibody was removed before nuclear counterstaining with DAPI at room temperature. Fluorescence images were acquired by confocal laser scanning microscopy (CLSM) and analyzed using ImageJ.

### 2.14 Motion capture

We observed nanomotors movement using Nanosight NS500. The system recorded trajectories at 5 frames per second under both X-ray irradiated and non-irradiated conditions, with ImageJ software processing the positional data. For speed calculations, we analyzed trajectories from at least five replicate videos, randomly selecting two fields of view per recording. Each field contained approximately 10 particles, yielding 10 tracked nanomotors per experimental group for mean velocity determination.

The mean-square-displacement (MSD) of GM-R848 nanomotors was calculated by using the following: MSD(Δt) = < ∑_{i=x, y}_[r_i_(t + Δt) − r_i_(t)]^2^ >, where < > is averaged over 10 nanoparticles, where r is a two-dimensional vector, i is an index to show x and y, and Δt represents the time interval.

The effective diffusion coefficient (D_eff_) was determined using: D_eff_ = MSD/(4Δt), where MSD is the mean squared displacement and Δt denotes the time interval. The average speed (v) was determined using: v = s/t, where s is the total path length traveled over time t. For each condition, measurements were obtained from at least 10 nanomotors.

### 2.15 Transwell test

To investigate nanomotors permeability in tumors, we seeded fibroblasts (1 × 10^5^ cells mL^−1^) as a monolayer in Transwell upper chambers to model tumor tissue. The fibroblasts were incubated with GM-R848-FITC (50 μg/mL) with or without X-ray irradiation. Then the CT26 cells in the lower chamber were stained with DAPI (5 μg/mL, 10 min) for nuclear labeling and imaged via confocal laser scanning microscopy (CLSM, 20× objective) at specified time points (10, 30, 60 min).

### 2.16 *In vitro* construction of CT26-derived multicellular spheroids (MCSs)

CT26 cells were seeded onto ultra-low adhesion culture plates to form spheroids. Cells were treated with GM-R848-FITC (50 μg/mL) with or without X-ray irradiation (2 Gy) and incubated for 4 h. The spheroids were subsequently examined using CLSM.

### 2.17 Advanced CRC model

Female BALB/c mice (6 weeks old) were obtained from Hunan SJA laboratory animal co., LTD. Animals and the procedures (2025PS1337K) were approved by the China Medical University Animal Care and Use Committee, and all animal experiments obeyed the rules. We established an advanced CRC model using BALB/c mice. Each mouse was injected intraperitoneally (i.p.) with 2 × 10^5^ CT26 cells suspended. After allowing 7 days for tumor growth, we administered intraperitoneal injections of PBS (50 μL), GM (50 μL, 5 mg/kg), or GM-R848 (50 μL, 5 mg/kg) on days 7 and 14. Tumor areas received 2 Gy X-ray irradiation 1 h post-injection. The mice underwent monitoring of body weight and abdominal circumference for 32 days, with survival rates recorded for each group (n = 6). To further evaluate the therapeutic effect, we collected ascites via abdominal puncture from each group on day 21, and measured ascites volume and tumor weight (n = 5).

### 2.18 Immunohistochemical staining analysis

For Ki-67 immunostaining, tumor samples were fixed, dehydrated in graded ethanol, and embedded in paraffin. Tissue sections from each experimental group underwent deparaffinization and rehydration by baking slides at 90 °C for 1.5 h, followed by xylene treatment and sequential ethanol washes (100 %, 90 %, and 70 %) before distilled water immersion. Antigen retrieval was performed using microwave heating with EDTA or citrate buffer, after which sections were blocked with 1 % BSA for 15 min at room temperature. Immunostaining employed a Ki-67 antibody (1:500 dilution), and positive cell quantification involved random field selection of equal size followed by ImageJ analysis.

### 2.19 RNA isolation, cDNA synthesis, and reverse transcription-quantitative polymerase chain reaction (RT-qPCR)

Peritoneal cells were isolated from lavage fluid of mice and subjected to red blood cell lysis. Total RNA was then extracted from the peritoneal cells using Trizol reagent according to the manufacturer's instructions. After quantification, 2 μg of total RNA was treated with gDNA Eraser and reverse-transcribed using the PrimeScript RT reagent Kit (42 °C for 50 min; 85 °C for 5 min). The qPCR reaction mixture was prepared according to the instructions of the TB Green Premix Ex *Taq*II (Tli RNaseH Plus) kit. The Real-Time PCR amplification was performed using the LightCycler® 96 real-time fluorescence quantitative PCR system. The expression of each target gene was normalized to the endogenous control β-actin, and relative fold changes were calculated using the comparative 2^−ΔΔCt^ method. All primer sequences are provided in Supplementary Table S1.

### 2.20 Statistical analysis

Statistical analysis was performed using GraphPad Prism 10.0 software, and values were expressed as the mean ± standard deviation (SD). Statistical analysis of variance (ANOVA) followed by Tukey's multiple comparisons test was used to compare differences between different treatment groups. Differences were regarded as statistically significant with ∗*p* < 0.05, ∗∗*p* < 0.01, ∗∗∗*p* < 0.001.

## 3 Results and discussion

### 3.1 Preparation and characterization of GM-R848 nanomotors

**Fig. 1:**
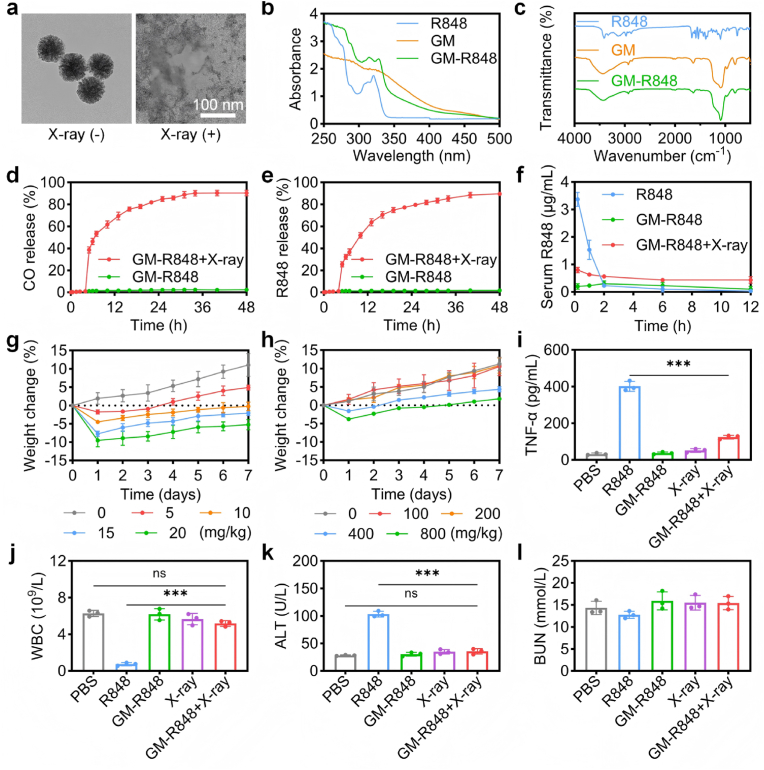
Synthesis, characterization and X-ray-responsive drug release behavior of GM-R848. (a) TEM images of GM-R848 that were incubated in PBS solutions for 24 h with X-ray irradiation (2 Gy). (b) UV–vis spectra and (c) FT-IR spectra of R848, GM, and GM-R848. (d) Cumulative release of CO and (e) R848 from GM-R848 with or without X-ray irradiation (2 Gy) (n = 3). (f) Plasma levels of R848 after intraperitoneal administration of free R848 (10 mg/kg) and GM-R848 (800 mg/kg) with or without X-ray irradiation (2 Gy) (n = 3). Weight loss measurements for mice following intraperitoneal administration of increasing concentrations of (g) free R848 and (h) GM-R848 (n = 3). (i) The serum cytokine levels of TNF-α taken at 2 h following intraperitoneal administration of free R848 (10 mg/kg) and GM-R848 (80 mg/kg) with or without X-ray irradiation (2 Gy) (n = 3). (j) WBC count, (k) liver enzyme alanine aminotransferase (ALT) and (l) blood urea nitrogentaken (BUN) taken at 24 h following intraperitoneal administration of free R848 (10 mg/kg) and GM-R848 (80 mg/kg) with or without X-ray irradiation (2 Gy) (n = 3). ∗∗∗*p* < 0.001.

The synthesis of gas nanomotors was achieved by first preparing an FeCO-bridged organosilane precursor FeCO-Si through a ligand-exchange reaction, as previously reported [37], and illustrated in Fig. S1. Following this, gas nanomotors (GM-R848) capable of stabilizing unstable FeCO prodrugs and encapsulating R848 for the X-ray-driven co-release of CO and R848 were fabricated using a modified sol-gel approach (Fig. S2). TEOS and FeCO-Si served as the silica precursors in this process. Transmission electron microscopy (TEM) and scanning electron microscopy (SEM) confirmed the monodisperse spheroidal morphology of the GM-R848 nanomotors, with an average diameter of 50 nm (Fig. 1a–S3 and S4). High-angle annular dark-field scanning transmission electron microscopy (HAADF-STEM) and energy dispersive X-ray spectroscopy (EDX) provided further confirmation of the co-localization of Fe, S, and Si within the nanomotors framework (Figs. S5 and S6), signifying the homogenous distribution of the FeCO-bridged organosilane within the structure. Additionally, ultraviolet–visible (UV–vis) spectroscopy revealed characteristic absorption peaks at 280 and 330 nm, confirming the co-presence of FeCO and R848 in the GM-R848 nanomotors (Fig. 1b) [37,38]. Fourier transform infrared (FT-IR) spectra, exhibiting νCO bands at 2000 cm^−1^, further substantiated the presence of FeCO within the GM-R848 nanomotors. The disappearance of R848's characteristic peak in purified GM-R848 confirms successful encapsulation and core localization of the compound (Fig. 1c) [38].

The GM-R848 nanomotors demonstrated a high gas storage capacity, with a CO content of 3.51 μmol/mg. The loading degree (LD) of R848 (12.50 ± 0.72 %) was determined by ultraviolet (UV) absorption spectroscopy, as quantified using the calibration curve (Fig. S7). These substantial gas reserves provide a continuous and robust source of energy for gas-driven propulsion. The Brunauer-Emmett-Teller (BET) surface area of the GM-R848 nanomotors was determined to be 375.09 m^2^/g, with an average pore diameter of 5.76 nm (Fig. S8). The rough surface structure facilitates the adsorption and subsequent release of CO molecules, providing numerous active sites for CO bubble formation and gas propulsion. Moreover, the mesoporous framework with its large pores enhances molecular exchange, enabling the rapid release of encapsulated drug molecules [39,40].

To examine the X-ray-driven release profile, GM-R848 nanomotors were dispersed in PBS solution with or without X-ray irradiation (2 Gy). As depicted in Fig. 1a and S4, exposure to X-rays induced the collapse of GM-R848 into irregular aggregates, which further disintegrated into smaller fragments within 24 h. This behavior is likely due to the high-energy X-rays breaking the Fe-CO bonds, thereby promoting the degradation of the nanomotors framework [37]. In contrast, in the absence of X-ray irradiation, GM-R848 demonstrated excellent stability in both PBS and fetal bovine serum (FBS)-containing media over a 72-h period (Fig. S9). Notably, GM-R848 nanomotors exhibited minimal spontaneous release (<2 % for both CO and R848 without X-ray irradiation), whereas X-ray irradiation (2 Gy) triggered the sustained co-release of CO and R848 over the course of 24 h (Fig. 1d–e and S10). A sequential release profile was observed, with CO being released more rapidly than R848. This phenomenon is attributed to the initial X-ray-induced decomposition of FeCO, leading to the breakdown of the GM-R848 framework and the subsequent release of the encapsulated R848.

### 3.2 Spatiotemporal control release to minimizes systemic toxicity

Resiquimod (R848), a small-molecule agonist of toll-like receptors 7 and 8, is recognized for its immunostimulatory properties, particularly its ability to activate innate immunity and alleviate immunosuppression [41,42]. However, the systemic administration of R848 is often associated with rapid dissemination from the injection site, leading to a pronounced systemic inflammatory response that limits its clinical utility [43,44]. The maximum tolerated dose (MTD) of R848 and GM-R848 was evaluated by monitoring body weight changes and clinical signs (body condition score) for one week. Mice treated with doses of 10 and 15 mg/kg exhibited signs of restricted mobility, lethargy, and weight loss exceeding 5 % of their initial body weight, establishing 10 mg/kg as the MTD (Fig. 1g). In contrast, GM-R848 demonstrated a significantly higher MTD of 800 mg/kg, approximately ten times greater than that of free R848 (Fig. 1h).

To assess the potential improvement in safety, we investigated whether the X-ray-triggered release of R848 in GM-R848 influenced the inflammatory response. Serum and intraperitoneal fluid (IP fluid) were collected 2 h after intraperitoneal injection and analyzed for cytokines typically released downstream of TLR7/8 activation. Notably, at equivalent R848 doses, GM-R848 significantly reduced systemic cytokine levels (Fig. 1i and S11) and maintained physiological white blood cell (WBC) counts (Fig. 1j), whereas free R848 induced a substantial systemic cytokine response and caused leukopenia--reflecting lymphocyte migration out of the bloodstream [45]. These findings suggest that GM-R848 significantly mitigates systemic inflammation. This differential response is likely attributable to the pharmacokinetics of R848 release (Fig. 1f and S12). The controlled release profile of GM-R848 thus minimizes systemic toxicity, as evidenced by the normalization of key inflammatory biomarkers (Fig. 1k–l and S13). Subsequently, systemic safety of GM-R848 was evaluated by measuring blood carboxyhemoglobin (COHb) levels in mice. GM-R848 gradually exhibited a release pattern, peaking in concentration within 24 h and returning to baseline levels by 72 h (Fig. S14). In conclusion, GM-R848 nanomotors represent a robust codelivery platform, facilitating X-ray-triggered, spatiotemporally precise release of both CO and R848. This controlled release significantly reduces systemic toxicity, enhancing the therapeutic safety profile of R848.

### 3.3 Synergistic strategy for radio-immunostimulatory

**Fig. 2:**
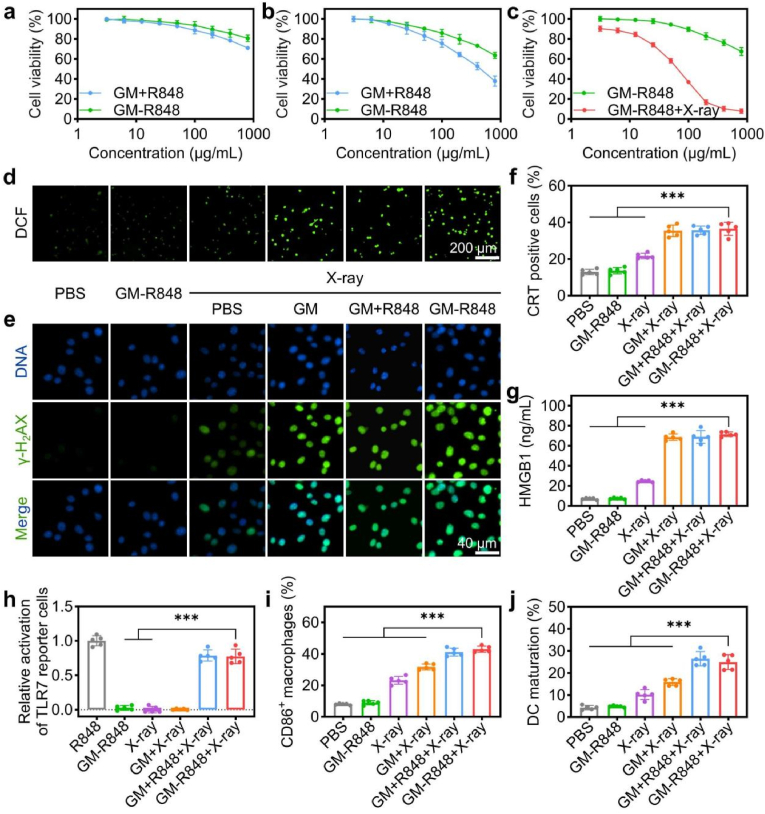
Antitumor efficacy and immunogenic cell death induced by GM-R848 with X-ray activation. (a) The viability of HIEC-6 cells and (b) Raw264.7 after incubation with GM + R848 and GM-R848 (n = 5). (c) The viability of CT26 cells after incubation with GM-R848 with or without X-ray irradiation (2 Gy) (n = 5). (d) Fluorescent images of CT26 cells after various treatments and stained with DCFH-DA probe for detecting intracellular ROS generation (n = 5). (e) Fluorescent images of CT26 cells after various treatments and stained with anti-γ-H_2_AX antibody for detecting intracellular DNA double-strand breaks (n = 5). (f) Percentage of CRT-positive cells and (g) amount of released HMGB1 of CT26 cells after various treatments (n = 5). (h) Relative activation of TLR7 reporter cells after various treatments (n = 5). (i) Percentage of CD86^+^ in CD11c^+^MHCII^+^ cells and (j) CD86^+^ macrophages after co-incubation with CT26 cells in different treatment groups (n = 5). ∗∗∗*p* < 0.001.

Given the spatiotemporally controlled release of CO and R848 from GM-R848 nanoparticles, we assessed their combinatorial therapeutic efficacy *in vitro* via MTT viability assay. As demonstrated in Fig. 2a–c and S15, a 2 Gy dose of X-ray irradiation was sufficient to achieve complete degradation of GM-R848 without causing any observable toxic effects on HIEC-6 cells. GM-R848 exhibited high cell viability in HIEC-6 cells, RAW 264.7 murine macrophages, and CT26 cells, indicating excellent biocompatibility. As anticipated, X-ray treatment further enhanced the apoptotic effect of GM-R848 on CT26 cells, supporting the efficacy of X-ray-activated therapy. To investigate the underlying mechanisms, CO and ROS levels were assessed following different treatments using FL-CO-1 probe and DCFH-DA. Stronger green fluorescence signals were observed in the cells treated with GM-R848 + X-ray irradiation than in those treated with GM-R848 alone (Fig. S16). While X-rays alone generated a modest increase in ROS due to radiation therapy, GM-R848 with X-ray stimulation triggered a robust upregulation of intracellular ROS (Fig. 2d and S17). This marked increase in ROS production is attributed to the large amounts of CO released from GM-R848 upon X-ray irradiation [46,47]. This increase in ROS correlates with elevated DNA double-strand breaks, as evidenced by enhanced phosphorylated H_2_AX (γ-H_2_AX) levels (Fig. 2e and S18), revealing a synergistic mechanism wherein CO release from GM-R848 potentiates ROS generation during radiation therapy.

We further assessed immunogenic cell death (ICD) markers in CT26 cells following GM-R848 + X-ray treatment. GM-R848 + X-ray irradiation treatment significantly induced immunogenic cell death (ICD) in CT26 cells, characterized by increased cell surface exposure of calreticulin (CRT) and HMGB1 release compared to PBS controls (Fig. 2f–g and S19). Additionally, HEK-Blue hTLR7 reporter cells treated with GM-R848 exhibited significantly enhanced TLR7 signaling upon X-ray exposure, indicating efficient R848 release within the cells (Fig. 2h and S20). This activation of TLR7 is known to stimulate antigen-presenting cells (APCs), including dendritic cells (DCs) and macrophages, thereby promoting immune responses [[48], [49], [50], [51]].

We hypothesize that DAMPs released from treated tumor cells interact with R848 to co-stimulate APCs, particularly DCs, facilitating antigen presentation to T cells. To evaluate DC maturation, we treated bone marrow-derived dendritic cells (BMDCs) with conditioned media from CT26 cells exposed to GM-R848 and X-ray irradiation. Flow cytometry analysis revealed that GM-R848 + X-ray irradiation significantly increased the proportion of matured DCs (mDCs) (Fig. 2i–j and S21-22). Correspondingly, GM-R848 + X-ray-treated CT26 cells secreted significantly higher levels of IL-6 and TNF-α (11.5- and 19.5-fold increases, respectively) compared to GM-R848 alone, and 4.4- and 7.0-fold higher levels compared to X-ray treatment alone (Fig. S23). Finally, to evaluate the antigen presentation capabilities of GM-R848 + X-ray treatment, we stimulated BMDCs with supernatants from CT26-OVA cells (CT26 cells transfected with ovalbumin antigen) for 24 h and co-cultured them with OT-I T cells (OVA-specific T cells) for an additional 48 h. GM-R848 + X-ray treatment resulted in a significant increase in IFN-γ production by T cells, indicating enhanced antigen presentation (Fig. S24). These results suggest that the combination of DAMPs and pathogen-associated molecular patterns (PAMPs) induced by GM-R848 + X-ray treatment potentiates the presentation of tumor-associated antigens, promoting a more robust anti-tumor immune response.

### 3.4 Gas-propelled nanomotors for enhanced tumor penetration and retention

**Fig. 3:**
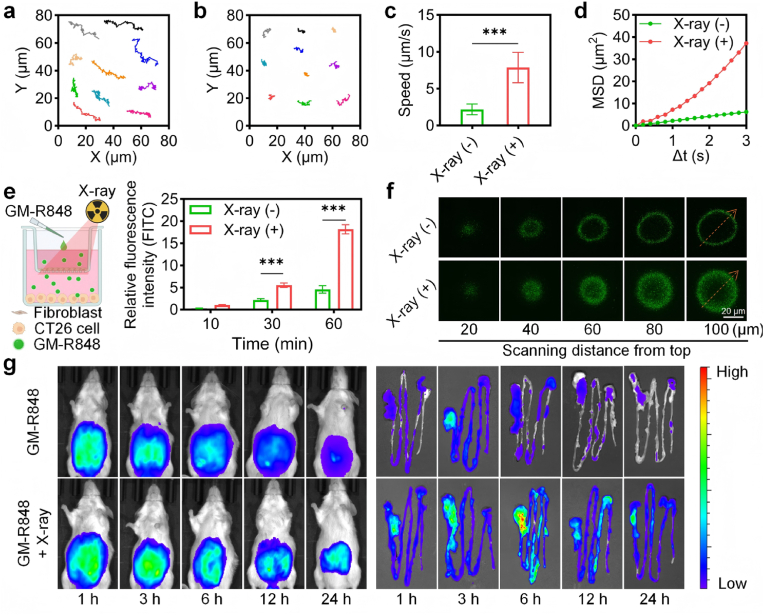
X-ray-driven motion behavior and deep tumor targeting of the GM-R848 nanomotors. (a) Trajectories of GM-R848 with or (b) without X-ray irradiation (2 Gy) for 10 s (n = 10). (c) The velocity of the GM-R848 nanomotor with or without X-ray irradiation (2 Gy) (n = 10). (d) The corresponding MSD of GM-R848 nanomotors with or without X-ray irradiation (2 Gy) (n = 10). (e) Schematic diagram of Transwell assays and relative fluorescence intensity measured in lower chamber CT26 cells by CLSM after incubation with GM-R84 with or without X-ray irradiation (2 Gy) for 10, 30 and 60 min (n = 3). (f) Z-stack CLSM images of GM-R848-FITC distributed in MTSs. (g) Fluorescence imaging of advanced CRC bearing mice and intestines at indicated time points after treatment with GM-R848-ICG with or without X-ray irradiation (2 Gy). ∗∗∗*p* < 0.001.

Following confirmation of CO generation from GM-R848 under X-ray irradiation, the autonomous motion of these nanomotors was characterized using the Nanosight NS500, with trajectories analyzed via ImageJ. Under X-ray irradiation, GM-R848 exhibited directed motion predominantly in a single direction, contrasting with the typical Brownian motion observed in the absence of X-ray exposure (Fig. 3a and b and Videos S1-S2). The average velocity of GM-R848 reached 7.89 μm/s upon X-ray irradiation, a 3.59-fold increase over its Brownian motion velocity (Fig. 3c). Additionally, the diffusion coefficient of GM-R848 under X-ray irradiation was 2.23 μm^2^/s, reflecting a 321 % enhancement compared to the non-irradiated state (Fig. S25). Analysis of mean square displacement (MSD) revealed a linear relationship with time intervals (Δt) for GM-R848 under X-ray irradiation (Fig. 3d), indicating that CO release significantly enhances diffusion efficiency through X-ray-triggered gas propulsion.

Supplementary data related to this article can be found online at https://doi.org/10.1016/j.mtbio.2025.102398

The following are the Supplementary data related to this article:

The impact of this propulsion on cellular uptake and penetration was subsequently evaluated. X-ray-irradiated GM-R848 demonstrated markedly increased intracellular fluorescence intensity, suggesting enhanced internalization facilitated by gas propulsion (Fig. S26). Transwell assays further assessed penetration across biological barriers, with fibroblasts cultured in the upper chamber and CT26 tumor cells in the basal chamber. FITC-labeled GM-R848 with X-ray irradiation exhibited significantly higher fluorescence in the basal chamber compared to non-irradiated controls (Fig. 3e and S27). To recapitulate the tumor microenvironment *in vitro*, CT26-derived multicellular spheroids (MCSs) were utilized as a three-dimensional model. FITC-labeled GM-R848, when subjected to X-ray irradiation, exhibited a significantly enhanced fluorescence signal at a depth of 100 μm within the MCSs compared to non-irradiated controls (Fig. 3f and S28). These results collectively demonstrate that X-ray-triggered gas propulsion enhances transcellular transport across biological barriers. Building on these *in vitro* findings, the *in vivo* distribution and intratumoral retention of GM-R848 were investigated in an advanced CRC model. ICG-labeled GM-R848 accumulation at the tumor site peaked 6 h post-injection, with over 30 % of the peak fluorescence intensity retained after 24 h (Fig. 3g and S29). Notably, ICG-labeled GM-R848 with X-ray irradiation exhibited a 5.14-fold higher fluorescence intensity compared to non-irradiated controls, underscoring the role of X-ray-triggered gas propulsion in enhancing tumor penetration and retention *in vivo*.

### 3.5 X-ray-activatable GM-R848 nanomotors for advanced CRC therapy

**Fig. 4:**
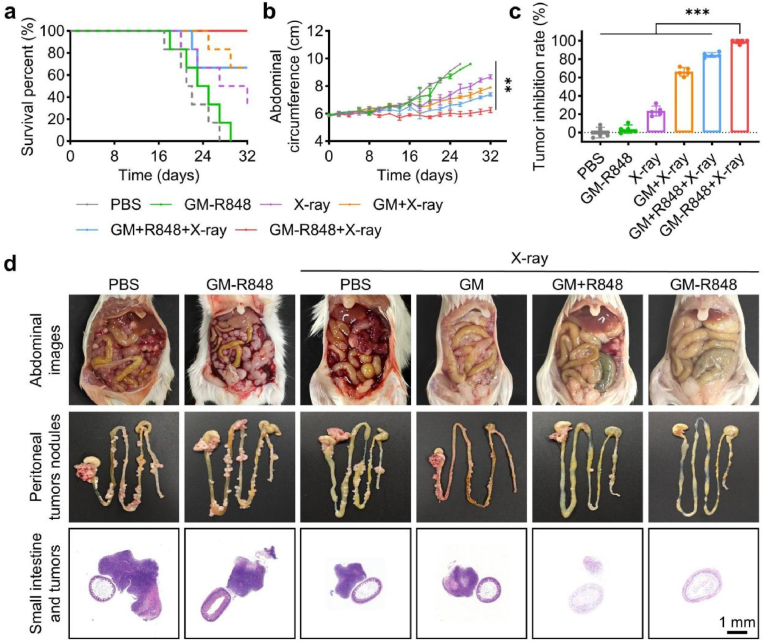
Therapeutic efficacy of GM-R848 against advanced CRC *in vivo*. (a) Survival curves of mice in different treatment groups for 32 days (n = 6). (b) Abdominal circumference of mice during the entire treatment period in different treatment groups (n = 6). (c) TIR of mice at 21 days in different treatment groups (n = 5). (d) Representative images of abdominal region, peritoneal tumors nodules and adhesive tumors which were stained with H&E at 21 days in different treatment groups. ∗∗*p* < 0.01, ∗∗∗*p* < 0.001.

Building on the potent anti-cancer and immunogenic cell death (ICD) effects observed *in vitro*, we evaluated the therapeutic efficacy of GM-R848 combined with X-ray irradiation in an advanced CRC model (Fig. S30). In the PBS-treated group, a marked increase in abdominal circumference was observed, indicative of extensive peritoneal seeding metastases and accumulation of malignant ascites (Fig. 4b). In contrast, mice treated with GM-R848 + X-ray exhibited no significant increase in abdominal circumference and achieved complete survival throughout the experimental period (Fig. 4a). To further assess therapeutic outcomes, selected mice from each group were euthanized on day 21 for analysis of ascites volume, tumor weight and nodule number (Fig. 4c–d and S31). The GM-R848 + X-ray treatment significantly reduced ascites volume and tumor burden, achieving a tumor inhibition rate (TIR) of 95.3 % relative to the PBS control. Notably, GM-R848 + X-ray outperformed the combination of GM + R848 + X-ray, likely due to the spatiotemporally controlled release of R848, which facilitated synergistic interplay between CO-mediated gas therapy and immunotherapy. Furthermore, these treatments don't induce significant histopathological abnormalities, injuries, or lesions compared to the control group (Fig. S32), indicating the absence of overt toxicity during treatments.

**Fig. 5:**
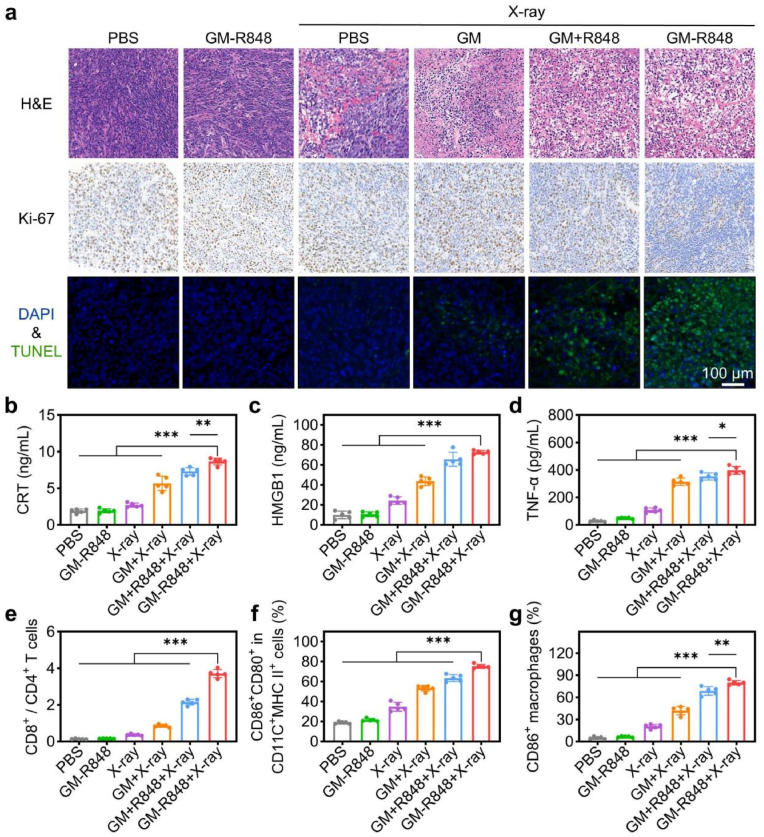
GM-R848 triggers immunogenic tumor elimination and activates antitumor immunity in malignant ascites. (a) H&E staining, Ki-67 immunohistochemistry staining and TUNEL staining of tumor sections in the different treatment groups. (b) The cytokine levels of CRT, (c) HMGB1 and (d) TNF-α in the ascites for different treatment groups. (e) The ratio of CD8^+^/CD4^+^ T cells, (f) CD80^+^CD86^+^ in CD11C^+^MHCII^+^ cells and (g) CD86^+^ macrophages in the ascites for different treatment groups (n = 5). ∗*P* < 0.05,∗∗*p* < 0.01, ∗∗∗*p* < 0.001.

Histological analysis via hematoxylin and eosin (H&E) staining of tumors treated with GM-R848 + X-ray revealed a high prevalence of apoptotic cells, characterized by nuclear chromatin pyknosis and cytoplasmic loss. Terminal deoxynucleotidyl transferase dUTP nick end labeling (TUNEL) staining further confirmed elevated levels of DNA fragmentation in apoptotic cells, while Ki67 staining indicated a significant reduction in tumor cell proliferation, underscoring the robust anti-tumor effects of GM-R848 + X-ray treatment (Fig. 5a and S33-34). To elucidate the mechanisms underlying these anti-tumor effects, we investigated immune cell infiltration in the ascites of advanced CRC model. Treatment with GM-R848 + X-ray induced a pronounced anti-cancer immune response, characterized by elevated levels of ICD markers (CRT and HMGB1) and pro-inflammatory cytokines (TNF-α, IL-6, and IFN-γ) (Fig. 5b–d and S35). Furthermore, GM-R848 + X-ray treatment significantly increased the infiltration of CD11c^+^MHCII^+^ DCs, elevated the proportion of CD8^+^ T cells, enhanced the CD8^+^ to CD4^+^ T cell ratio, and promoted the presence of M1-like macrophages in ascites (Fig. 5e–g and S36-38). These findings demonstrate that GM-R848 + X-ray treatment elicits robust anti-tumor immunity by engaging both innate and adaptive immune responses, driven by the synergistic effects of tumor antigen release through ICD and enhanced antigen presentation mediated by damage-associated molecular patterns DAMPs and PAMPs.

### 3.6 RNA-seq reveals molecular mechanisms underlying GM-R848 nanomotors therapy

**Fig. 6:**
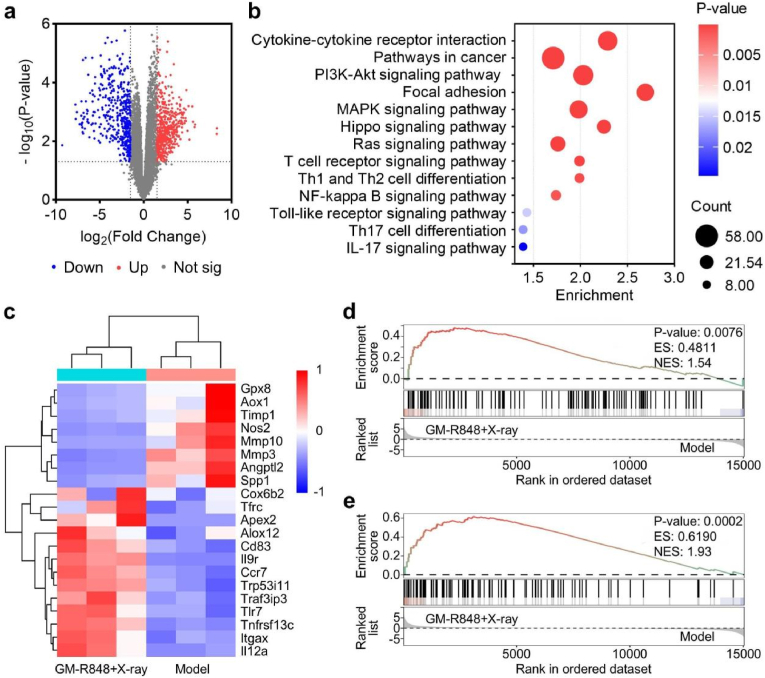
RNA sequencing analysis of the ascites in mice with advanced CRC under different treatment groups. (a) Volcano plots for all the expressed genes of Model versus GM-R848 + X-ray treatments. Red (upregulated) and Blue (downregulated) dots mean that the genes have significant differences (n = 3). (b) Significant enriched KEGG pathway terms of DEGs between Model group and the GM-R848 + X-ray group (n = 3). (c) The heatmap of representative differentially expressed genes between the Model group and the GM-R848 + X-ray group (n = 3). (d) GSEA enrichment plots of differentially expressed genes centralized in T cell receptor signaling pathway and (e) NF-kappa B signaling pathway between Model group and the GM-R848 + X-ray group (n = 3). (For interpretation of the references to color in this figure legend, the reader is referred to the Web version of this article.)

To elucidate the molecular mechanisms by which GM-R848 synergizes with X-ray irradiation to suppress tumorigenesis, we performed RNA sequencing (RNA-seq) analysis. Genes exhibiting a log_2_(fold change) of ≥1.5 were classified as upregulated, and those with ≤ −1.5 as downregulated, relative to the untreated model group. In the GM-R848 + X-ray treatment group, 980 differentially expressed genes (DEGs, |log_2_FC| ≥ 1.5, p ≤ 0.05) were identified, comprising 443 upregulated and 536 downregulated genes, indicative of substantial transcriptomic reprogramming (Fig. 6a and S39). Kyoto Encyclopedia of Genes and Genomes (KEGG) pathway analysis revealed that cytokine-cytokine receptor interactions were the most significantly altered, alongside other cancer-related pathways, including Pathways in cancer, PI3K-Akt signaling, MAPK signaling, Toll-like receptor signaling, and Ras signaling (Fig. 6b). Heatmap analysis further demonstrated that GM-R848 + X-ray treatment significantly upregulated pro-oxidant genes (e.g., Alox12) while downregulating antioxidant genes (e.g., Aox1), suggesting a shift toward a pro-oxidative cellular state. This redox imbalance likely contributed to DNA damage, as evidenced by the upregulation of base-excision repair genes (e.g., Apex2) and apoptosis effectors (e.g., Trp53i11), supporting a mechanism driven by reactive oxygen species (ROS)-mediated apoptosis. Concurrently, immune activation genes (e.g., Itgax and Traf3ip3) were upregulated, while tumor progression-associated genes (e.g., Mmp10 and Angptl2) were suppressed, indicating a transition toward an anti-tumorigenic transcriptional profile (Fig. 6c). Gene set enrichment analysis (GSEA) further revealed enrichment of gene sets associated with T cell receptor signaling and NF-κB signaling pathways in the GM-R848 + X-ray group compared to the model group (Fig. 6d). To experimentally validate the transcriptomic profiles and further elucidate the underlying molecular mechanisms, we performed RT-qPCR analysis on a series of key genes implicated in the enriched pathways (Fig. S40). Collectively, these transcriptomic findings suggest that GM-R848 + X-ray treatment enhances therapeutic efficacy through ROS-dependent apoptotic pathways and immune activation, offering a promising framework for the treatment of advanced CRC.

## 4 Conclusion

We developed an innovative X-ray-activated GM-R848 nanomotor by integrating FeCO-bridged organosilica moieties into a mesoporous silica framework. The GM-R848 nanomotors exhibit superior CO stability, high gas storage capacity, and controlled CO release compared to conventional post-loading gas nanomotors. Upon X-ray irradiation, the FeCO framework rapidly decomposes, generating CO bubbles that propel nanomotors motility, significantly improving transport, penetration, and cellular uptake. This was demonstrated *in vitro* using monolayer tumor cell models, transwell assays with multilayered cells, and three-dimensional tumor spheroid models, where GM-R848 achieved 3.3-fold greater penetration than passive nanoparticles. *In vivo* studies in an advanced CRC model further confirmed enhanced tumor penetration and retention, with GM-R848 distributing extensively throughout the tumor, highlighting the efficacy of CO bubble-driven propulsion in overcoming biological barriers. In comparison with recently reported nanomotor systems [[52], [53], [54], [55]], our GM-R848 nanomotors demonstrate several unique features. Although previous designs have utilized strategies such as pH sensitivity, redox responsiveness, receptor mediation or NIR-II laser irradiation to enhance tumor penetration, they typically lack externally controllable activation. By contrast, our system is activated by clinically available X-ray irradiation, providing a precisely controllable and non-invasive external trigger. Furthermore, the synergistic effect of this radio-gas-immunotherapy combination highlights its potential to enhance both local tumor control and systemic anticancer immunity. The key innovation lies in X-ray-triggered “gas propulsion + immune activation” dual functionality. Propulsion overcomes delivery barriers, and synchronized CO/R848 release couples radiosensitization with localized immune stimulation. This platform advances the functionalization of gas delivery systems and provides new perspectives for gas-driven nanomotors in biomedical applications.

Overall, we established a novel radio-immunostimulatory strategy for advanced CRC treatment. GM-R848 nanomotors mitigate immune-related adverse events associated with systemic R848 administration through X-ray-triggered, controlled release. Within the tumor, GM-R848 enhances R848 accumulation while CO disrupts tumor metabolic symbiosis, amplifying X-ray-induced ROS generation to trigger ICD. The synergistic effects of tumor antigen release and TLR7/8 pathway activation promote systemic anti-tumor immunity by enhancing DCs maturation, M1 macrophage polarization, and cytotoxic T lymphocyte infiltration. This multi-level radio-gas-immunotherapy approach achieved a remarkable 95.3 % tumor growth inhibition in advanced CRC models, with prolonged survival and minimal systemic toxicity. The integration of R848 and CO gas delivery presents a promising cancer immunotherapy strategy, offering significant potential for enhancing clinical radiotherapy. Collectively, these findings underscore the transformative potential of X-ray-driven gas-propelled nanomotors for precision oncology and their prospects for clinical translation in advanced CRC management.

## CRediT authorship contribution statement

**Xin Zhao:** Project administration, Methodology, Investigation. **Huayi Sun:** Project administration, Methodology, Investigation. **Zikun Shen:** Investigation, Formal analysis. **Shaowen Wang:** Project administration, Methodology, Investigation. **Fangman Chen:** Resources, Project administration, Investigation. **Xiaochun Xie:** Project administration, Methodology, Investigation. **Shuhui Wang:** Resources, Project administration, Methodology, Investigation. **Yucen Zhang:** Methodology, Investigation, Data curation, Conceptualization. **Yan Guo:** Resources, Methodology, Investigation, Formal analysis. **Yidan Zhang:** Supervision, Project administration, Methodology, Investigation. **Quanxin Ning:** Supervision, Software, Project administration, Methodology, Funding acquisition. **Dan Shao:** Project administration, Methodology, Funding acquisition. **Hong Zhang:** Writing – review & editing, Supervision, Funding acquisition, Conceptualization.

## Declaration of competing interest

The authors declare that they have no known competing financial interests or personal relationships that could have appeared to influence the work reported in this paper.

## Data Availability

Data will be made available on request.
