# Supplementary material for: X-ray-driven nanomotor with enhanced penetration and retention for carbon monoxide-amplified radioimmunotherapy of advanced colorectal cancer
Source: Mater Today Bio. 2025 Oct 13;35:102398. doi: 10.1016/j.mtbio.2025.102398 (PMC12552560; doi:10.1016/j.mtbio.2025.102398)
Supplement: Multimedia component 1 [file mmc1.docx]

**Supporting Information for**

**X-ray-driven Nanomotor with Enhanced Penetration and Retention for Carbon Monoxide-Ampli****fied Radioimmunotherapy of Advanced Colorectal Cancer**

Xin Zhao^a,b,1^, Huayi Sun^a,1^, Zikun Shen^b,1^, Shaowen Wang^b^, Fangman Chen^b^, Xiaochun Xie^b^, Shuhui Wang^a^, Yucen Zhang^c^, Yan Guo^c^, Yidan Zhang^b^, Quanxin Ning^b,*^, Dan Shao^b,*^, Hong Zhang^a,*^

^a^ Department of General Surgery, Shengjing Hospital of China Medical University, Shenyang 110004, China

^b^ National Engineering Research Center for Tissue Restoration and Reconstruction, South China University of Technology, Guangzhou 510006, China

^C^ Department of Anesthesiology, The Fifth Clinical Medical College of Inner Mongolia Medical University, Hohhot 010110, China

^*^ Corresponding authors.

1. mail addresses:

quanxin_ning@outlook.com (Q. Ning),

stanauagate@outlook.com (D. Shao),

[haojiubujian1203@sina.cn](mailto:haojiubujian1203@sina.cn) (H. Zhang).

^1^ These authors contributed equally to this work.

**Materials**

Dodecacarbonyltriiron (Fe_3_(CO)_12_, 96%) was obtained from Xin Ding Pengfei Technology Development Co., Ltd (Beijing, China). Resiquimod (R848) was bought from Adamas (Shanghai, China). 3-mercaptopropyltriethoxysilane (MPTES), tetraethyl orthosilicate (TEOS), 3-aminopropyltriethoxysilane (APTES), dimethyl sulfoxide (DMSO), fluorescein isothiocyanate isomer (FITC), indocyanine green (ICG), cetyltrimethylammonium bromide (CTAB), phosphate buffer saline (PBS), hemoglobin (Hb), sodium dithionite were purchased from Sigma-Aldrich Co. (St Louis, MO, USA). The 2,7-Dichlorodihydrofluorescein diacetate (DCFH-DA) was obtained from Solarbio (Beijing, China). RPMI-1640, Dulbecco's modified Eagle medium (DMEM), fetal bovine serum (FBS), 0.25% trypsin-EDTA and 1% penicillin- streptomycin were purchased from Gibco Co., Ltd. (Carlsbad, CA, USA). The 4',6-diamidino-2-phenylindole (DAPI) was purchased from Beyotime (Shanghai, China) and used according to the protocols provided by the manufacturers. The CO probe (FL-CO-1) was purchased from ruixi Biological Technology Co., Ltd (Xian, China). The 3-(4,5-dimethylthiazol-2-yl)-2,5-diphenyltetrazolium bromide (MTT) was purchased from Dalian Meilun Biotechnology Corporation (Dalian, China). Anti-mouse H_2_AX antibody was purchased from Abcam Sangon Biotech Co., Ltd (Shanghai, China). Hematoxylin staining solution, hematoxylin differentiation solution, Terminal deoxynucleotidyl transferase dUTP Nick End Labeling (TUNEL) and Ki-67 immunohistochemistry antibody were purchased from Servicebio Technology (Wuhan, China). Immuofluorescence antibodies for flow cytometry analysis of immune cells were purchased from BioLegend (MA, USA), including anti-CD11c-PE, anti-CD80-D549, anti-MHCII-AF700, anti-CD86-APC, anti-CD45.2-Cy7, anti- CD8a-BV785, anti-CD4-AF700, anti-CD3-BV421, anti-CRT-AF647. Mouse interferon-γ (IFN-γ) enzyme linked immunosorbent assay (ELISA) Kit was purchased from Beijing CHENG ZHI KE WEI Biotechnology Co., Ltd., (Beijing, China). Mouse necrosis factor-α (TNF-α), interleukin-6 (IL-6), human CRT (Calreticulin) ELISA kit and high mobility group box 1 (HMGB1) enzymelinked immunosorbent assay (ELISA) kit were purchased from Elabscience Biotechnology Co., Ltd (Wuhan, China).

**
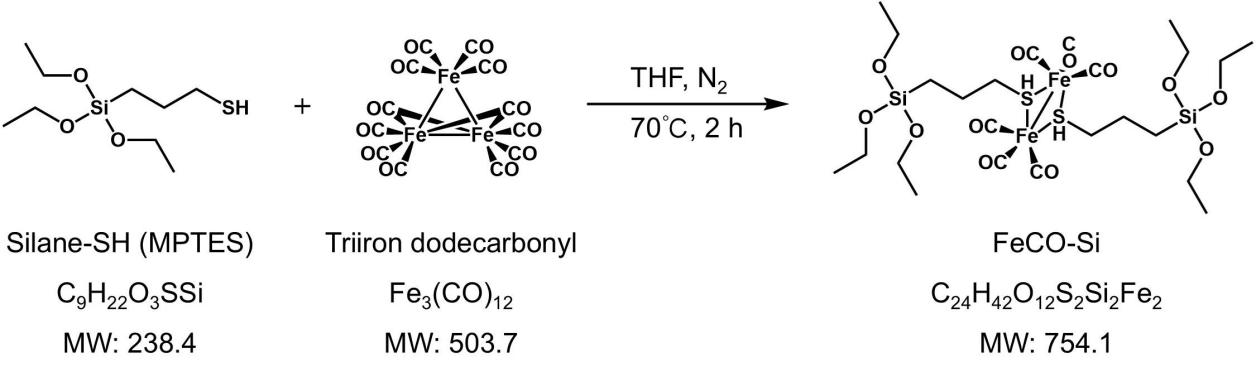
**

**Fig. S1.** Schematic diagram for the preparation of the FeCO-Si.


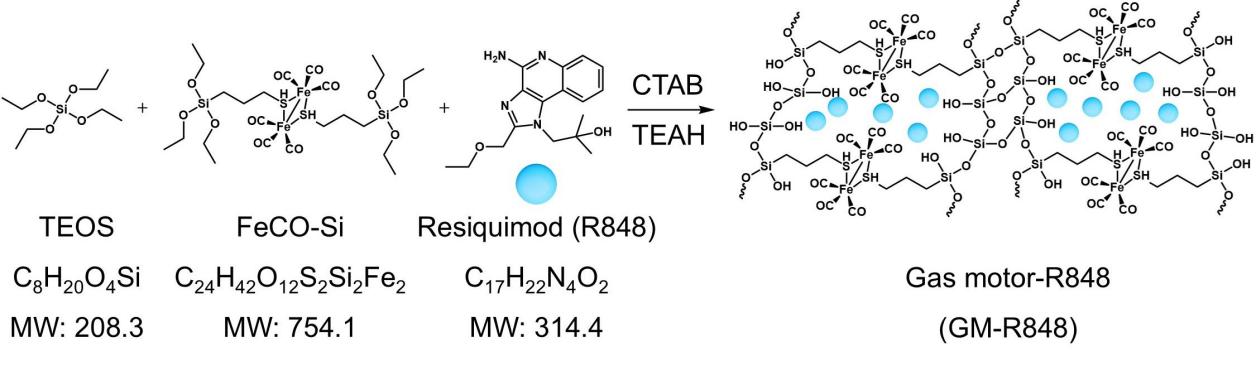


**Fig. S2.** Schematic diagram for the preparation of the GM-R848.


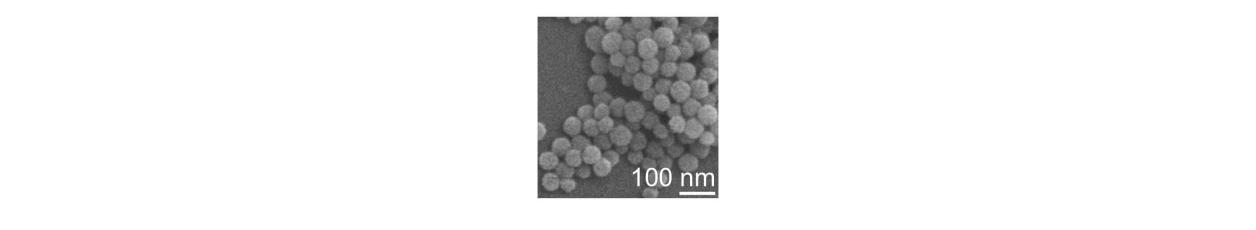


**Fig. S3.** SEM image of the GM-R848.


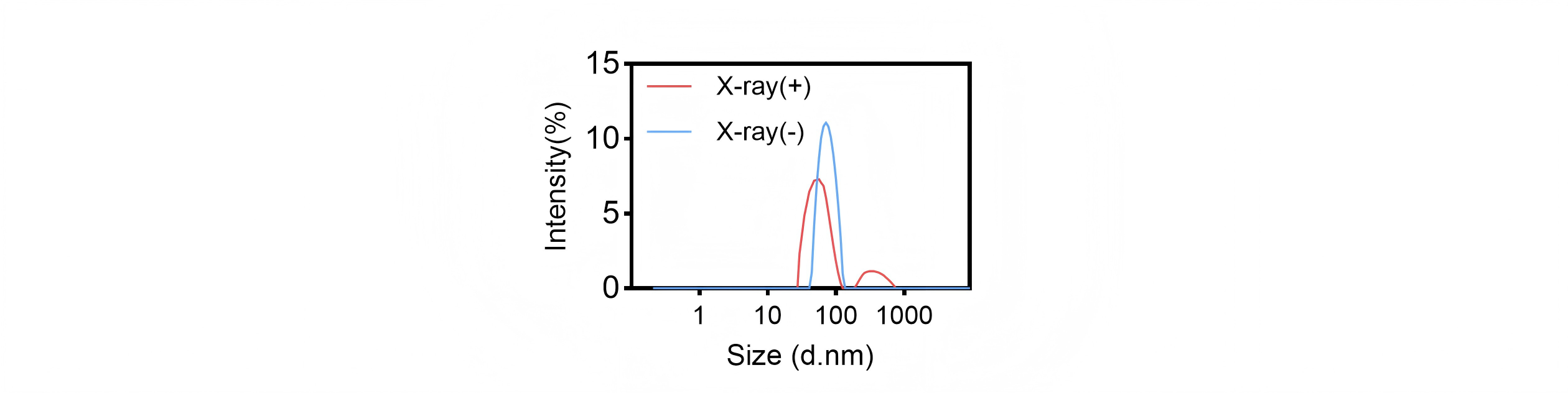


**Fig. S4.** Hydrodynamic size of the GM-R848 with or without X-ray irradiation (n = 5).


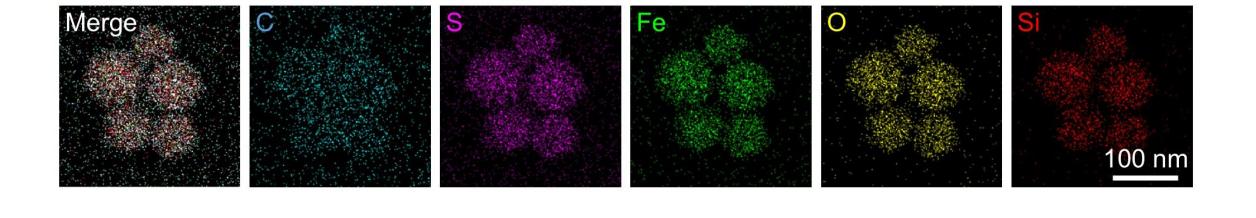


**Fig. S5.** HAADF-STEM images of the GM-R848.


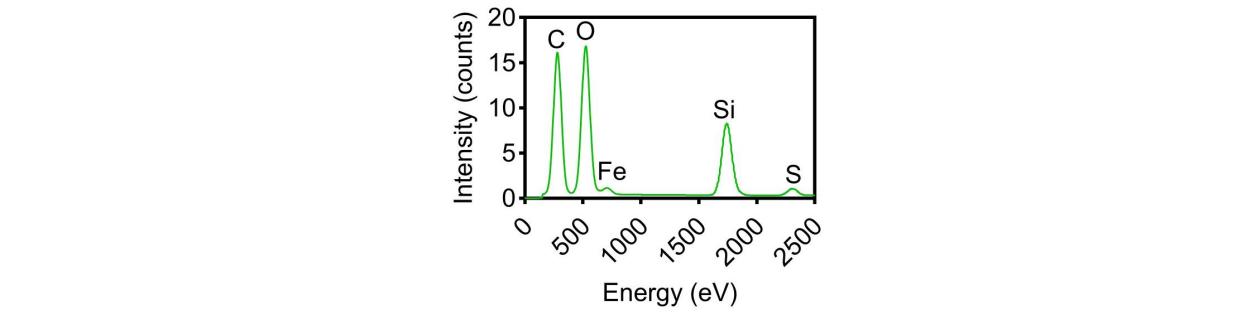


**Fig. S6.** EDX image of the GM-R848 obtained using TEM analysis.


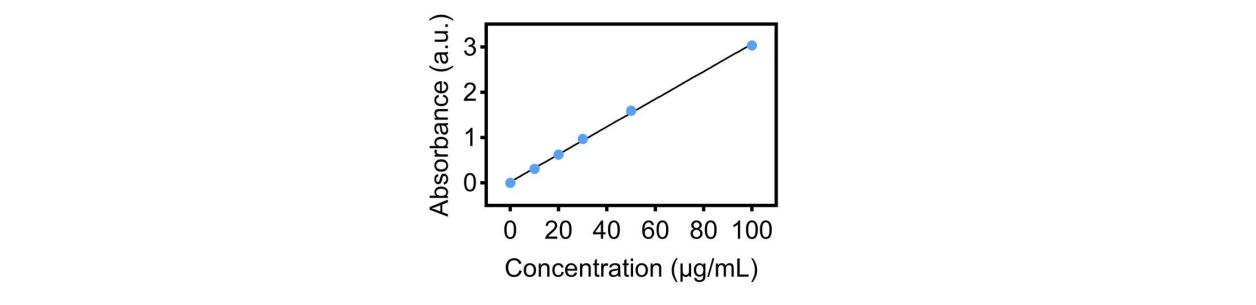


**Fig. S7.** The standard curve of R848 by UV-vis method. The data are expressed as the mean ± SD of three independent measurements (n = 3).


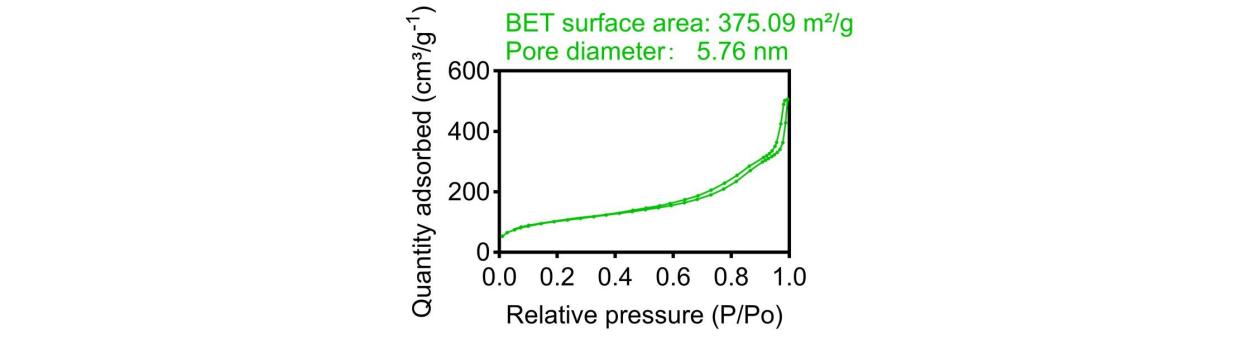


**Fig. S8.** N_2_ adsorption desorption isotherm of the GM-R848.


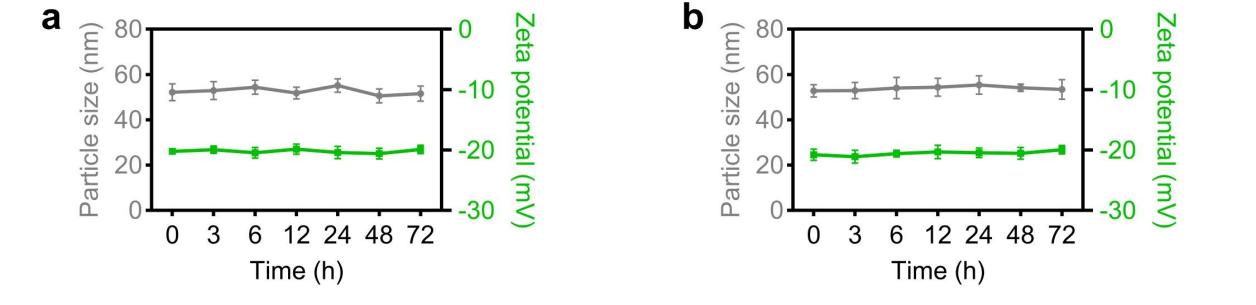


**Fig. S9.** Hydrodynamic size and zeta potential of GM-R848 in (a) PBS and (b) 10% FBS during 72 h determined by DLS test (n = 5).


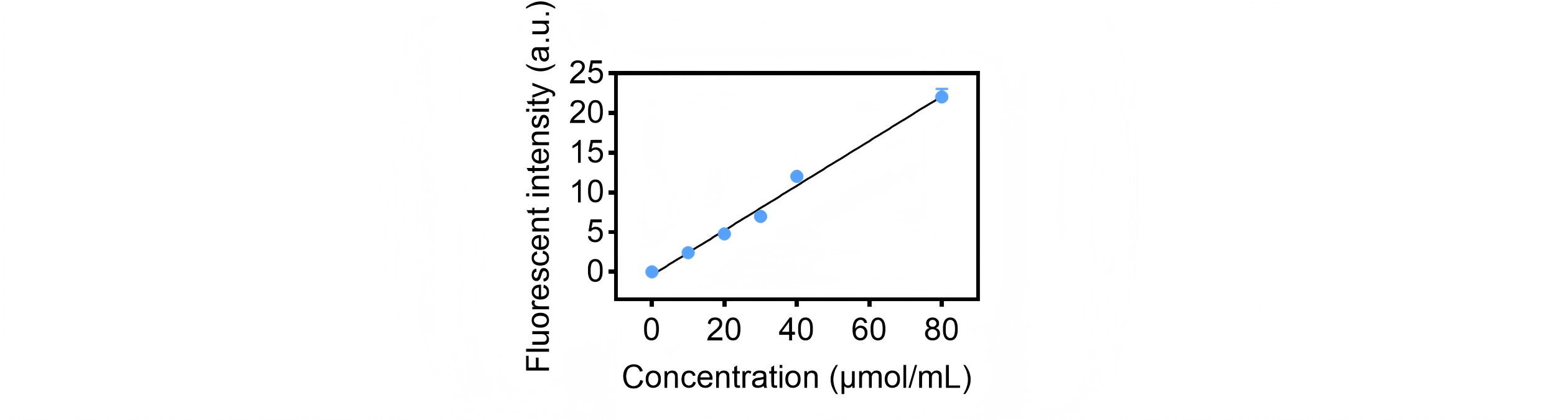


**Fig. S10.** The standard curve of CO by fluorescent intensity of FL-CO-1. The data are expressed as the mean ± SD of three independent measurements (n = 3).


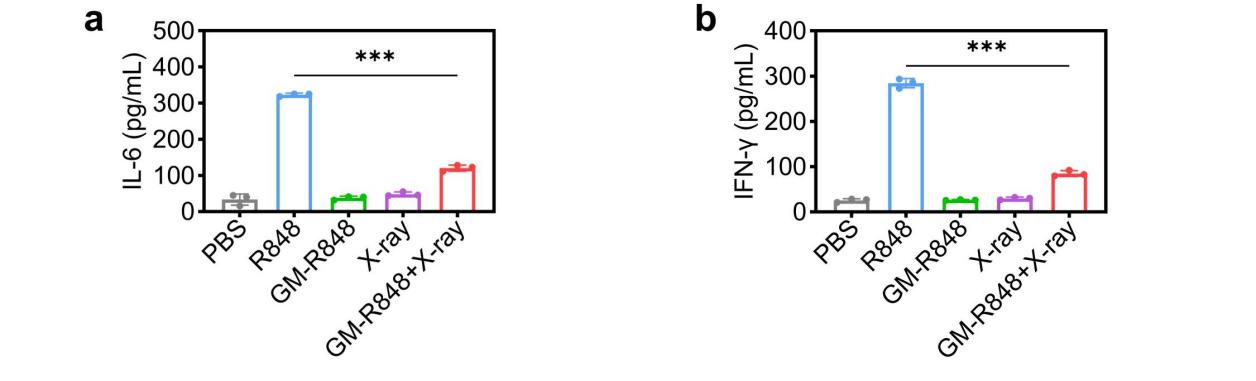


**Fig. S11.** The serum cytokine levels of (a) IL-6 and (b) IFN-γ taken at 2 h following intraperitoneal administration of free R848 (10 mg/kg) and GM-R848 (800 mg/kg) with or without X-ray irradiation (2 Gy) (n = 3). ^***^*p* < 0.001


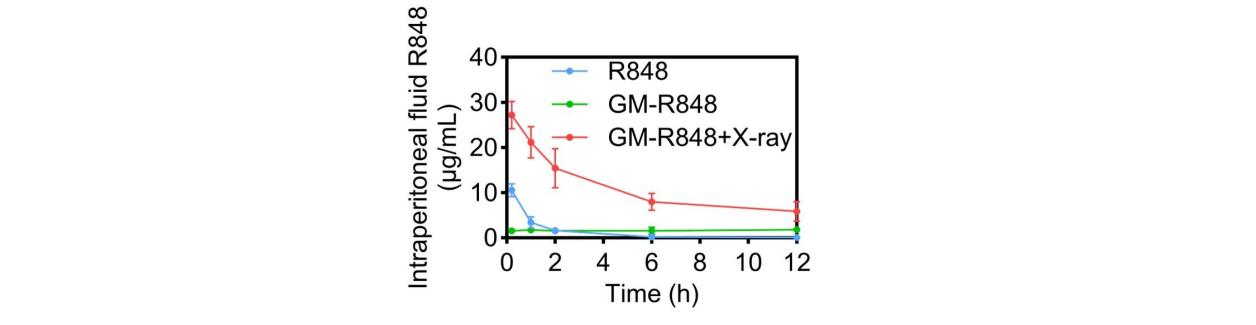


**Fig. S12.** Intraperitoneal fluid levels of R848 after intraperitoneal administration of free R848 (10 mg/kg) and GM-R848 (800 mg/kg) with or without X-ray irradiation (2 Gy) (n = 3).


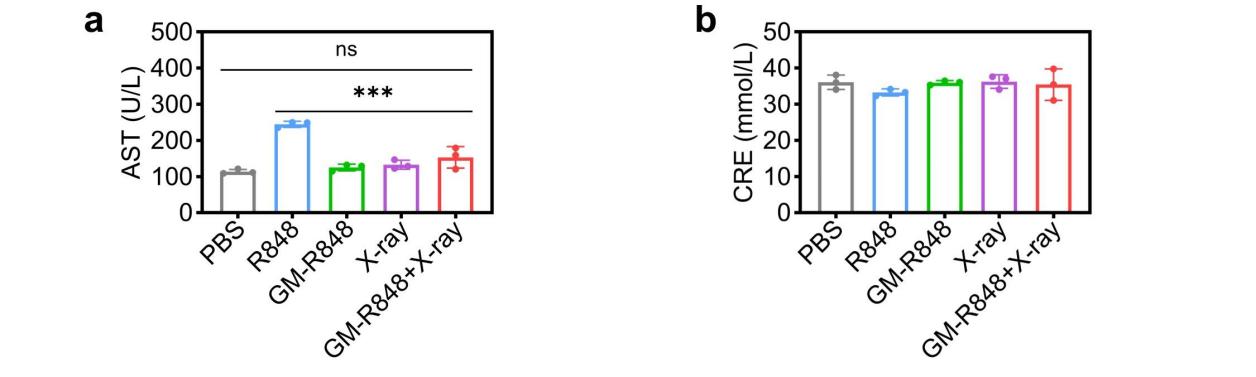


**Fig. S13.** Levels of (a) AST and (b) CRE taken at 24 hours following intraperitoneal administration of free R848 (10 mg/kg) and GM-R848 (800 mg/kg) with or without X-ray irradiation (2 Gy) (n = 3). ^***^*p* < 0.001


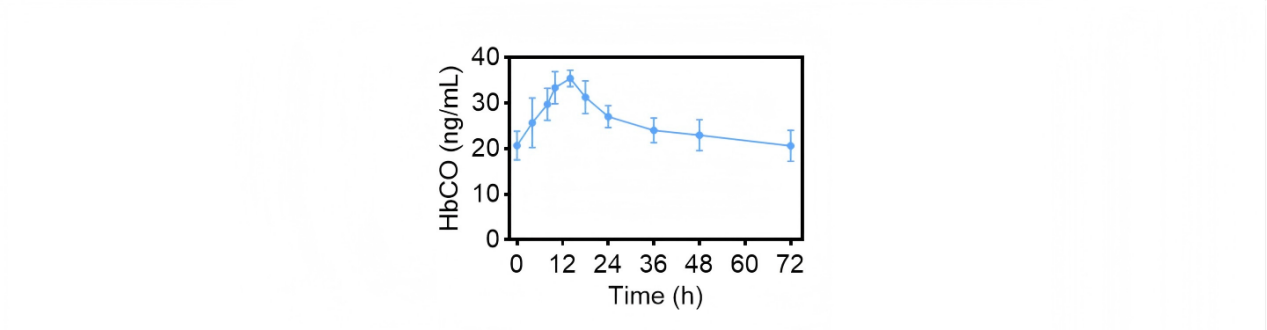


**Fig. S14.** Pharmacokinetics behavior of GM-R848 release in mice (n = 3).

*
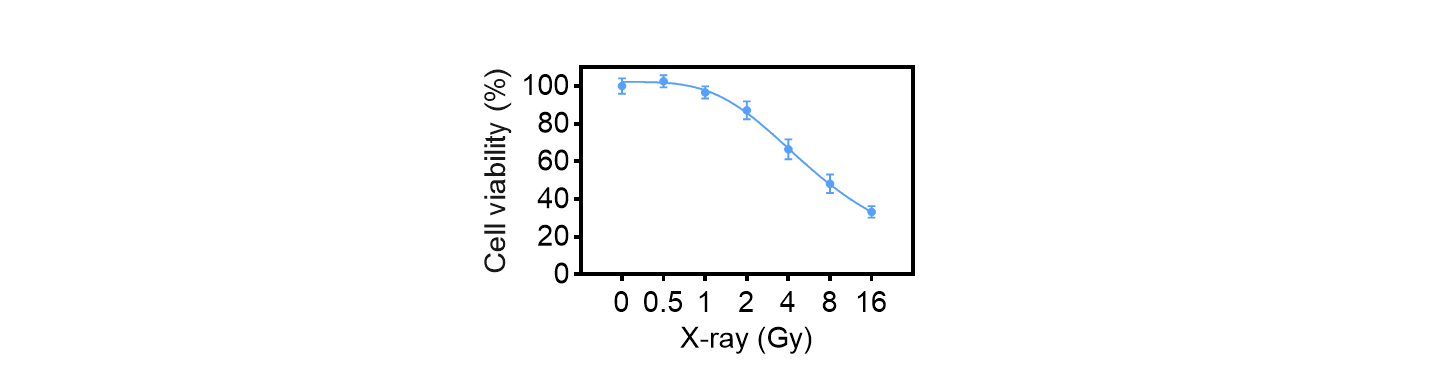
***Fig. S15.** The viability of HIEC-6 cells after incubation with X-ray irradiation (0-16 Gy) (n = 3).


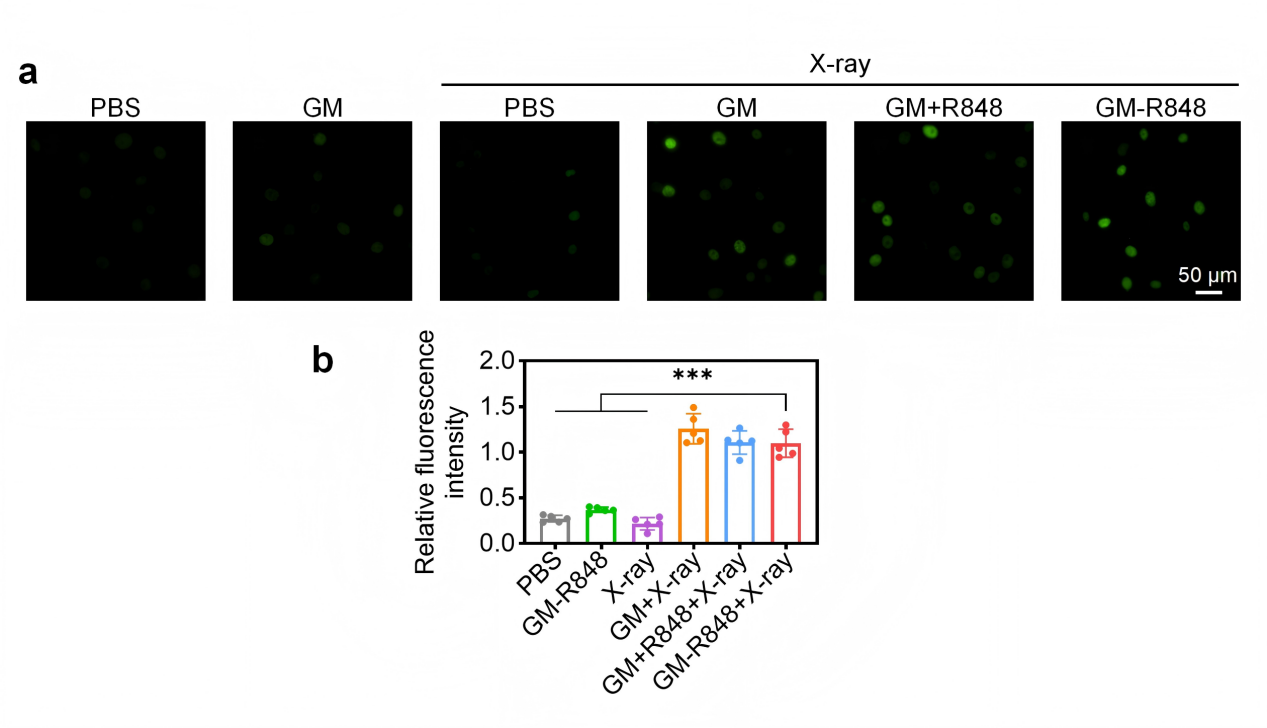


**Fig. S16.** Fluorescent images (a) and relative fluorescence intensity (b) of intracellular CO levels (detected via a FL-CO-1 probe using CLSM) at 24 h after various treatments (n = 5). ****p* < 0.001


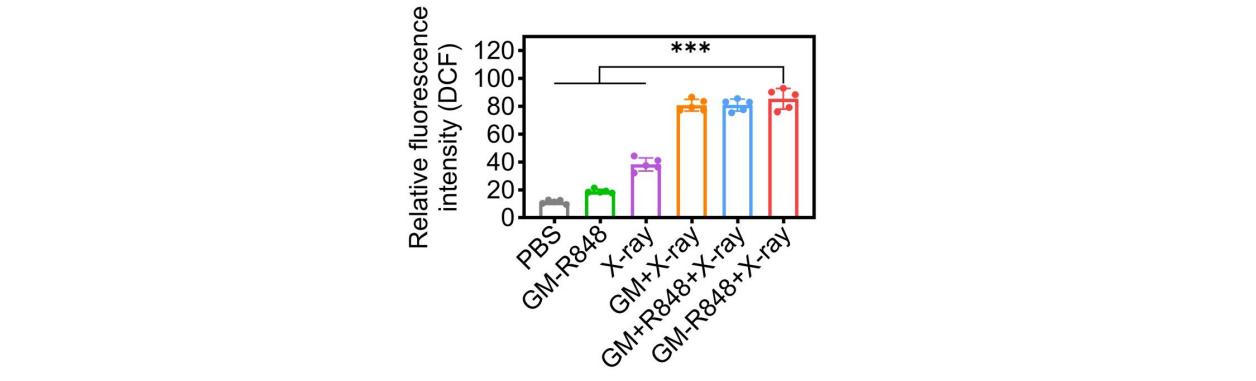


**Fig. S17.** Relative fluorescence intensity of CT26 cells after various treatments and stained with DCFH-DA probe for detecting intracellular ROS generation (n = 5). ^***^*p* < 0.001


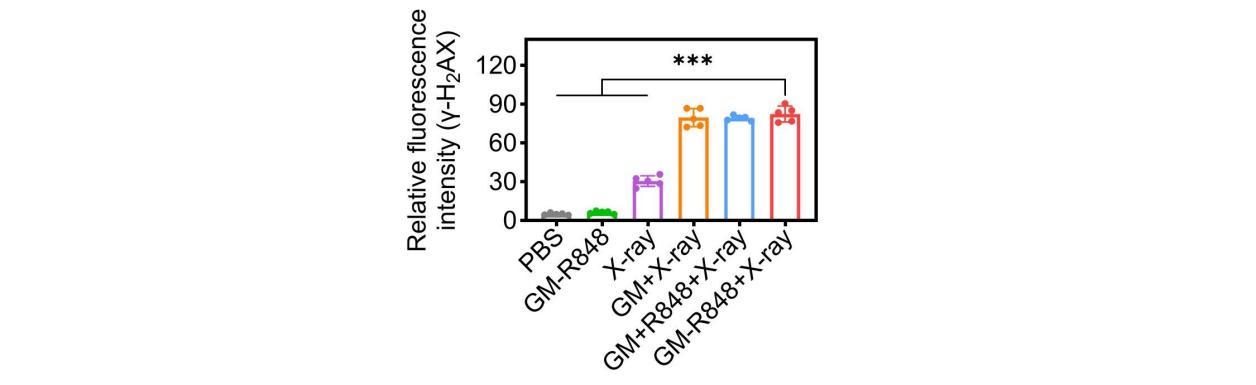


**Fig. S18.** Relative fluorescence intensity of CT26 cells after various treatments and stained with anti-γ-H2AX antibody for detecting intracellular DNA double-strand breaks (n = 5). ^***^*p* < 0.001


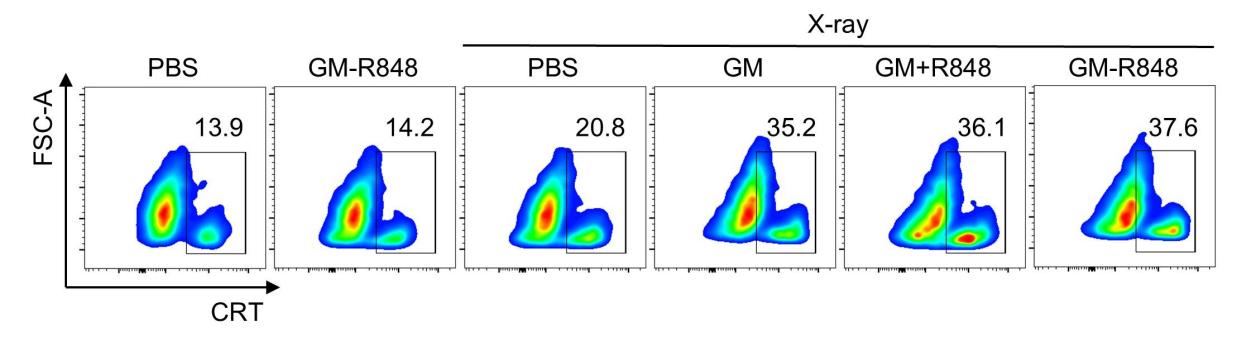


**Fig. S19.** Representative flow cytometry plots of CRT positive CT26 cells after various treatments (n = 5).


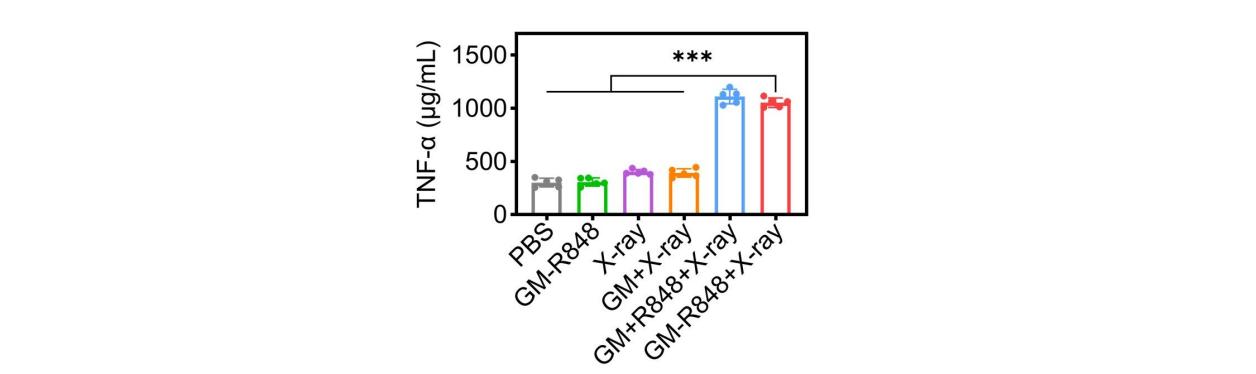


**Fig. S20.** The cytokine levels of TNF-α in supernatant of Raw264.7 cells after various treatments (n = 5). ^***^*p* < 0.001


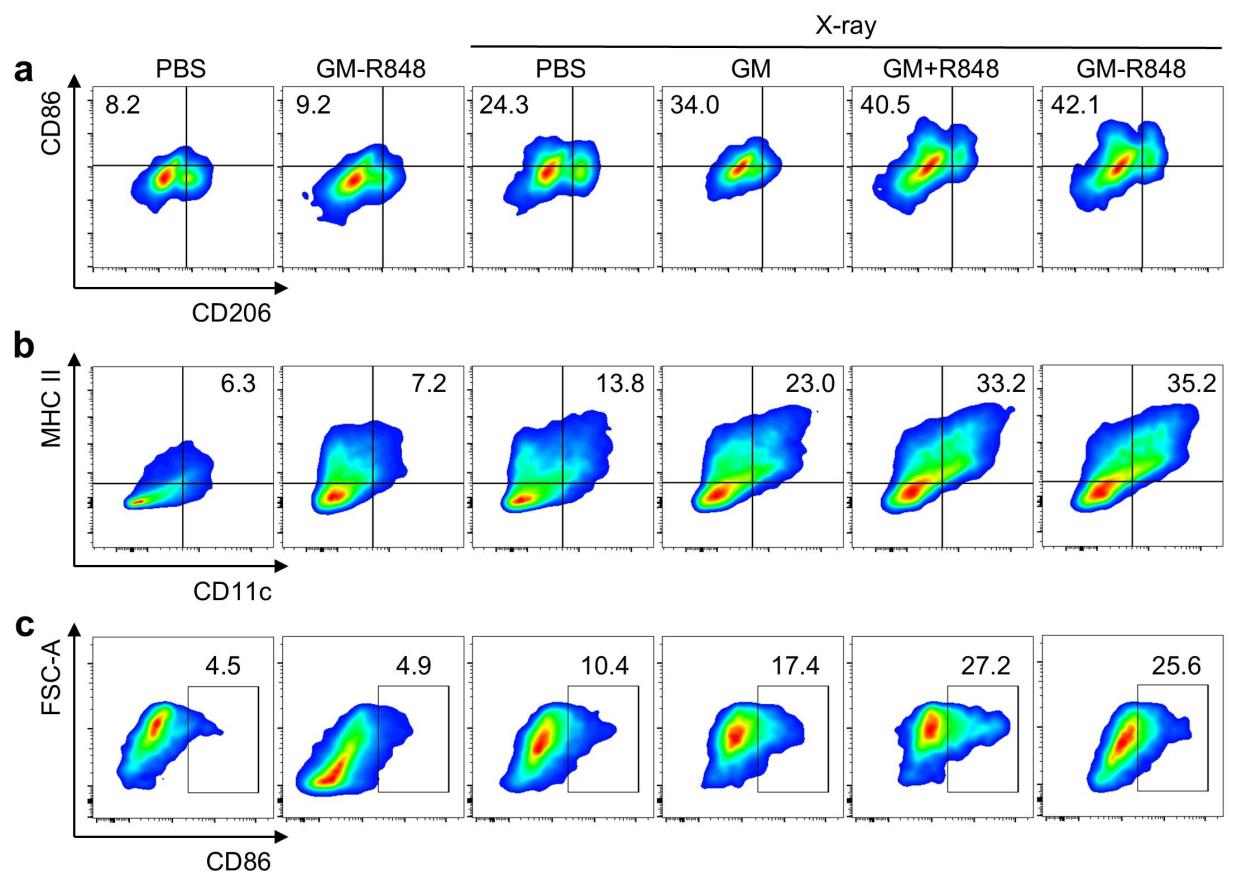


**Fig. S21.** Representative flow cytometry plots of (a) M1-like macrophages, (b) CD11c⁺MHCII⁺ antigen-presenting cells (APCs) and (c) CD86^+^ mature DCs after co-incubation with CT26 cells in different treatment groups (n = 5).


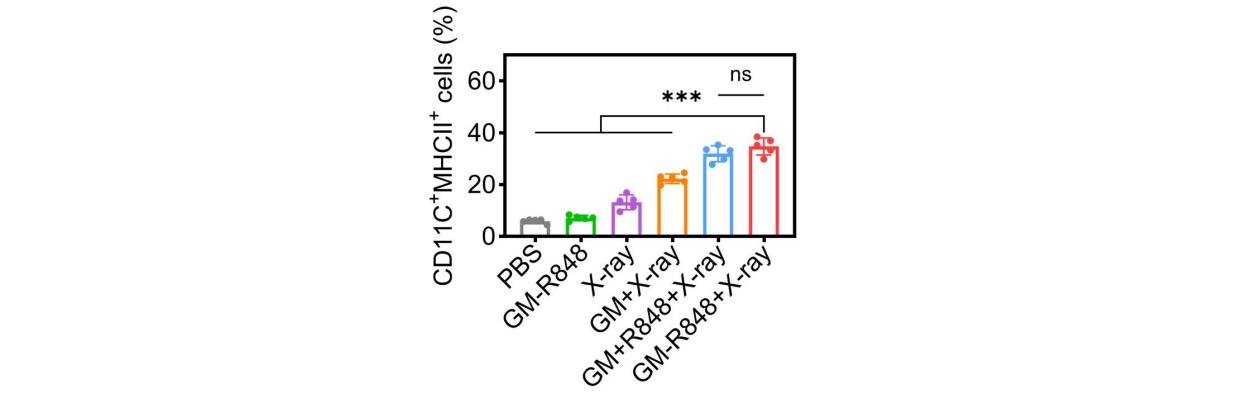


**Fig. S22.** Percentage of CD11c^+^MHCII^+^ cells after co-incubation with CT26 cells in different treatment groups (n = 5). ^***^*p* < 0.001


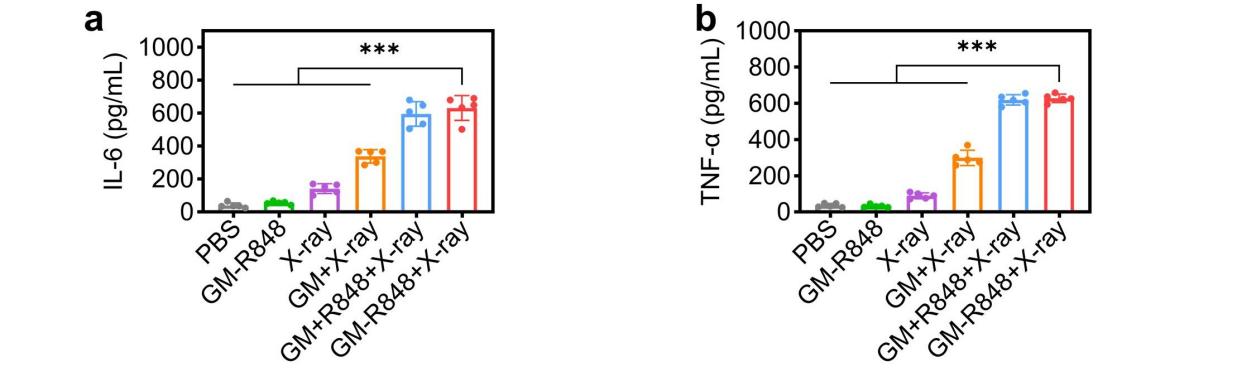


**Fig. S23.** The cytokine levels of (a) IL-6 and (b) TNF-α in supernatant of BMDCs after various treatments (n = 5). ^***^*p* < 0.001


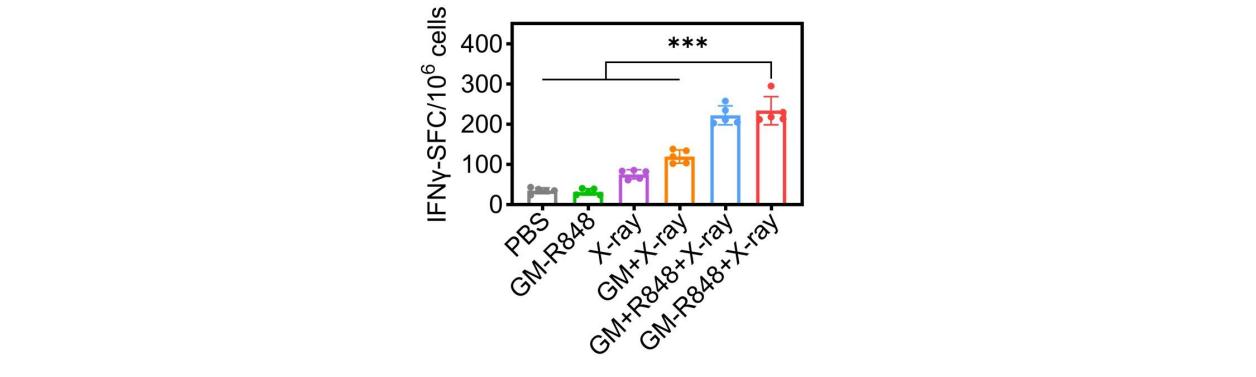


**Fig. S24.** Statistical analysis of ELISpot assay performed to detect tumor-specific IFN-γ-producing T cells. BMDCs were first stimulated with the supernatants of treated CT26-OVA (CT26 cells transfected with ovalbumin antigen) for 24 h, and then co-cultured with OT-I T cells (OVA-specific T cells) for another 48 h (n = 5). ^***^*p* < 0.001


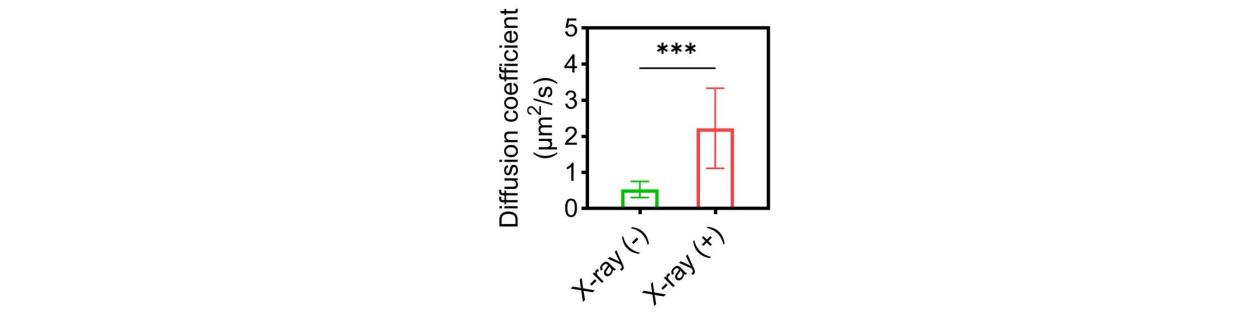


**Fig. S25.** The diffusion coefficients of the GM-R848 nanomotors with or without X-ray irradiation (2 Gy) (n = 10). ^***^*p* < 0.001


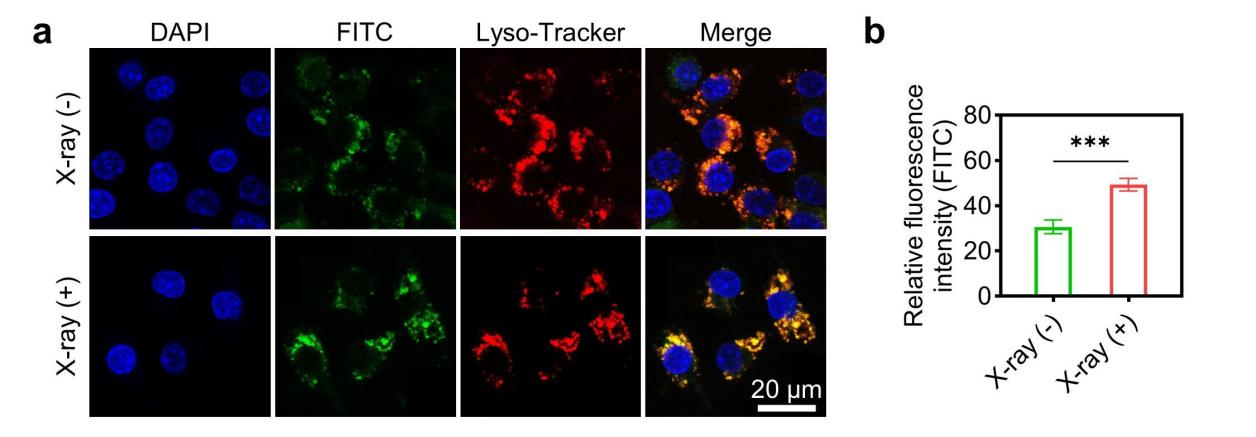


**Fig. S26.** Representative (a) fluorescent images and (b) relative fluorescence intensity of CT26 cells after treatment with GM-R848-FITC (green) for 4 h with or without X-ray irradiation (2 Gy). DAPI and Lyso-Tracker Red were used to stain cell nuclei (blue) and lysosomes (red), respectively (n = 3). ^***^*p* < 0.001


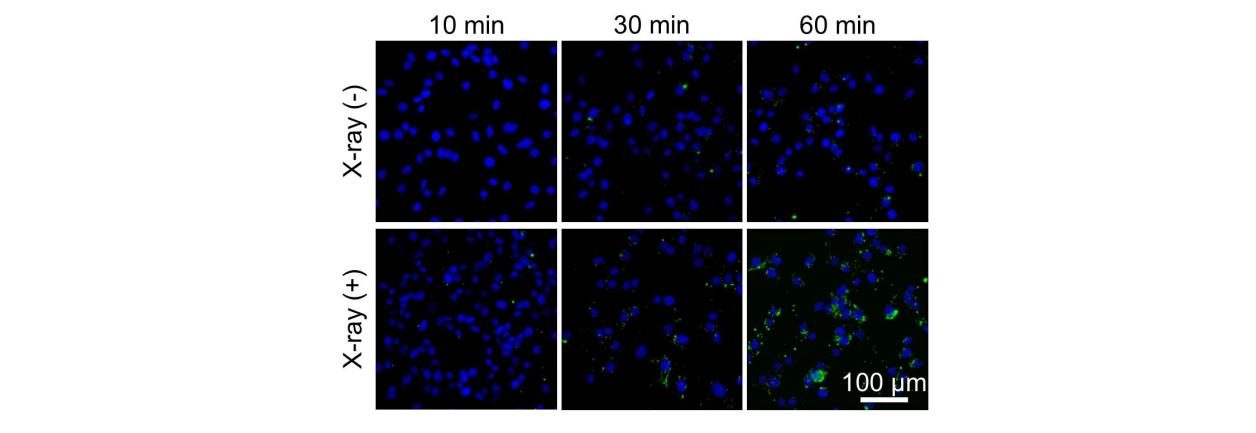


**Fig. S27.** Representative fluorescent images of lower chamber CT26 cells after incubation with GM-R848-FITC with or without X-ray irradiation (2 Gy) for 10, 30 and 60 min.


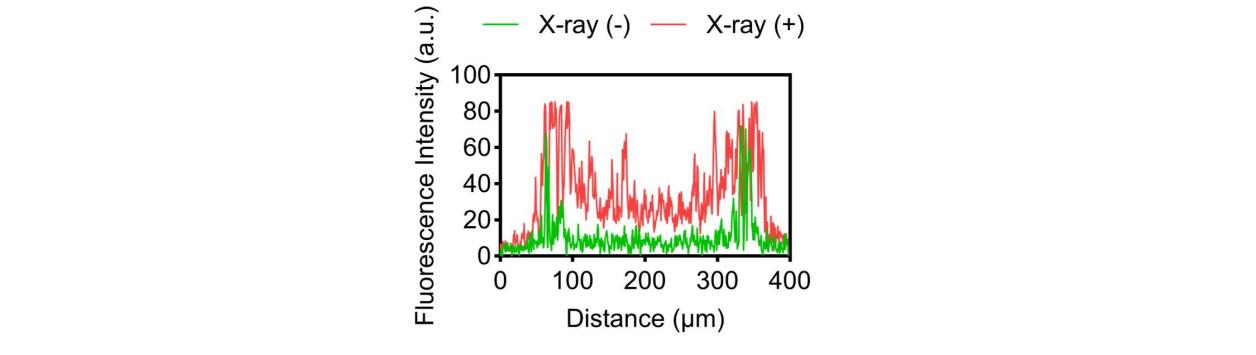


**Fig. S28.** Corresponding fluorescence distribution curves of Z = 100 μm in MCSs.


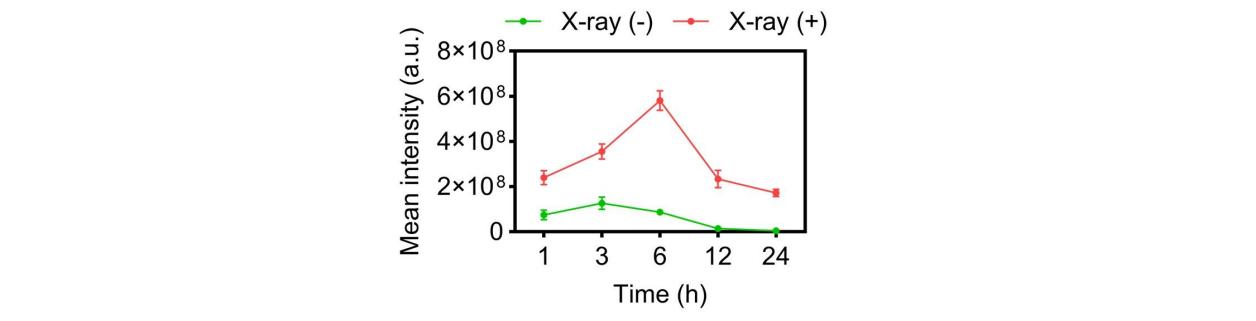


**Fig. S29.** Mean fluorescence intensity of intestines at indicated time points after treatment with GM-R848-ICG with or without X-ray irradiation (2 Gy) (n = 3).


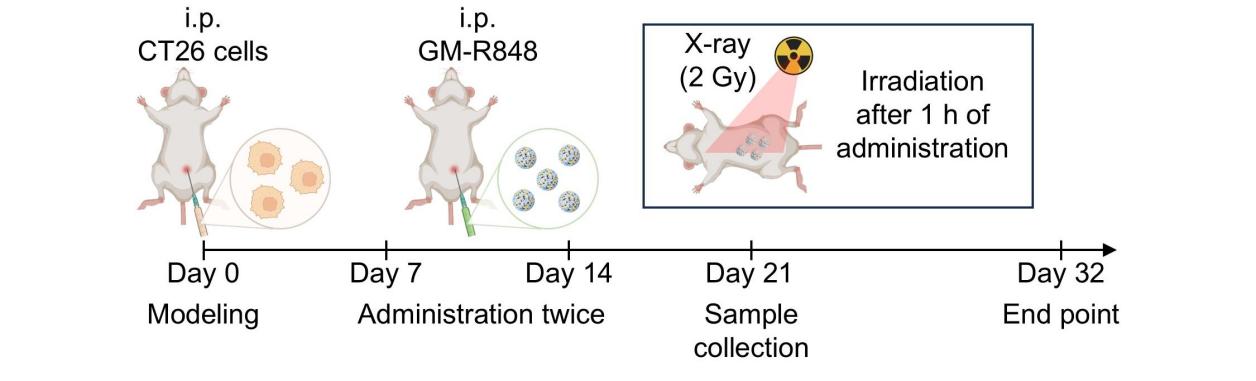


**Fig. S30.** Establishment of workflow and the experimental design in advanced CRC model.


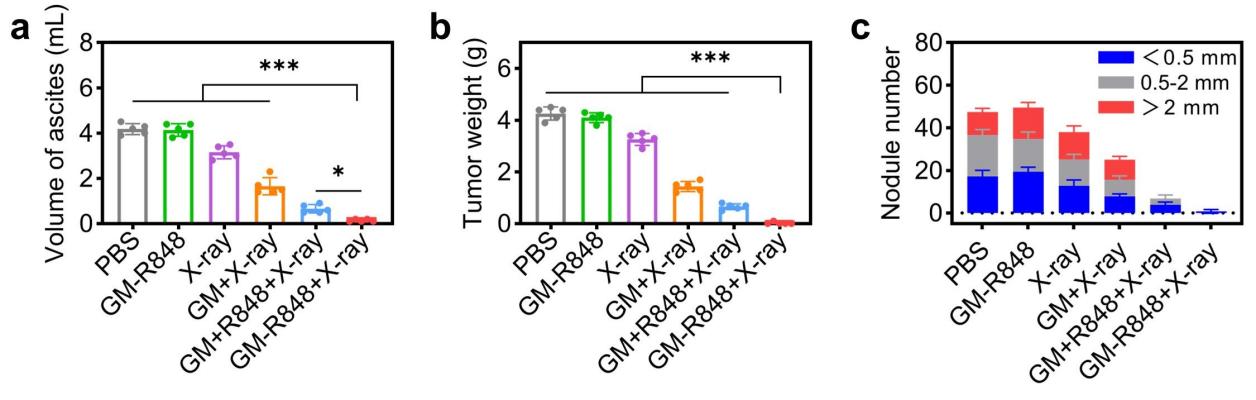


**Fig. S31.** (a) Ascites volume, (b) tumor weight and (c) nodule number of mice at 21 days in different treatment groups (n = 5). ^***^*p* < 0.001


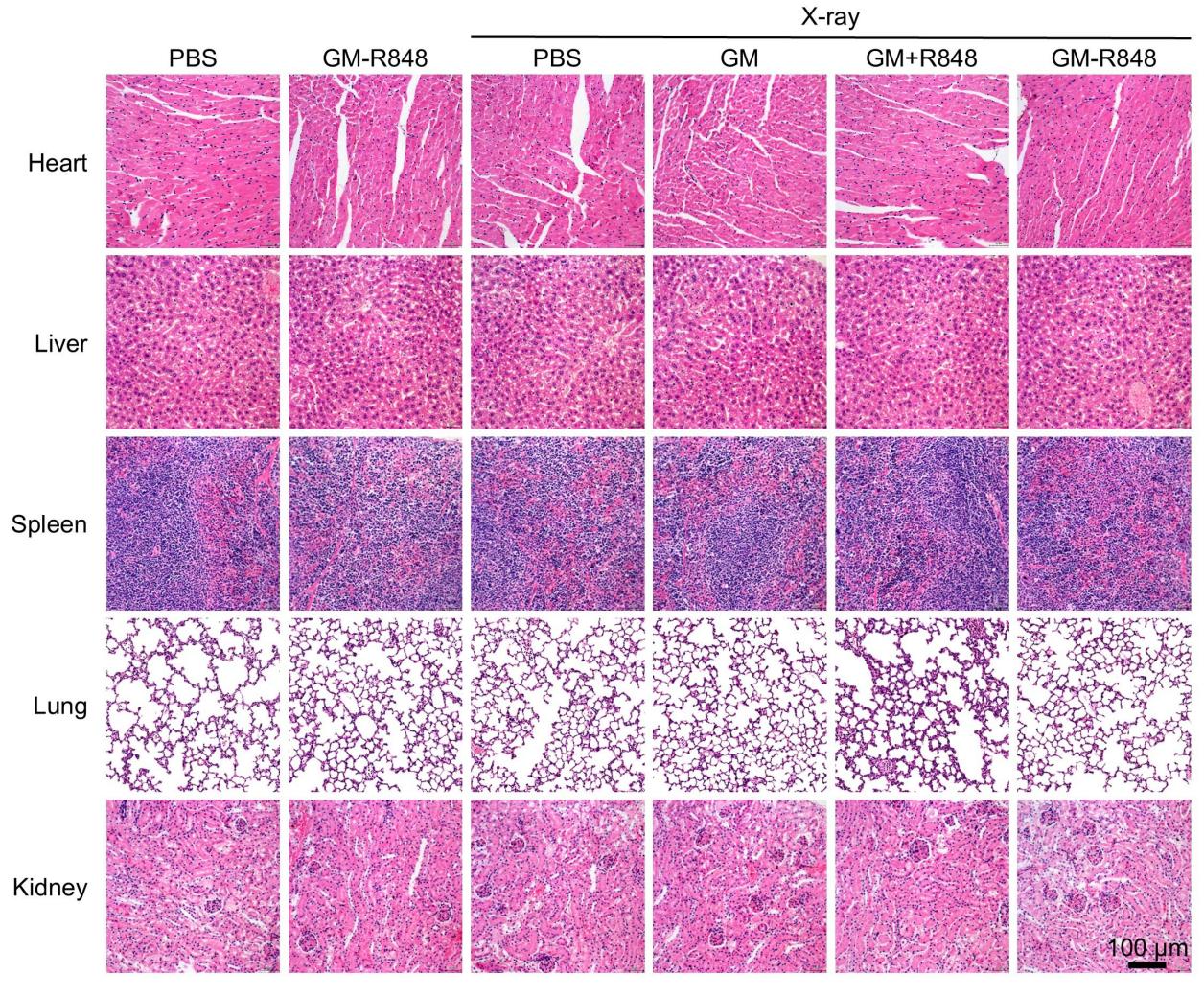


**Fig. S32.** Representative images of major organs (heart, liver, spleen, lung and kidney) were stained with H&E at the end of treatment in different treatment groups (n = 5).


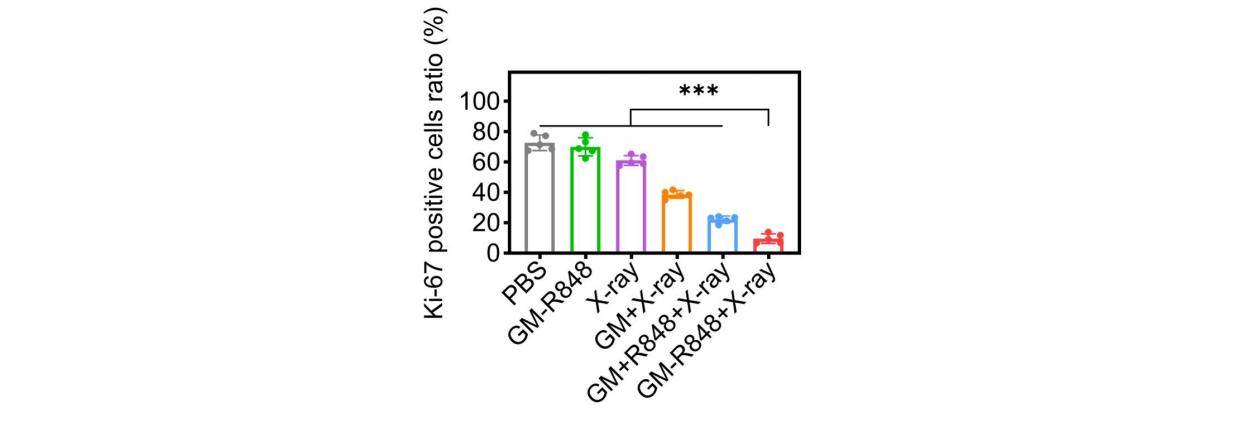


**Fig. S33.** Quantification of the expression levels of Ki-67 in different treatment groups (n = 5). ^***^*p* < 0.001


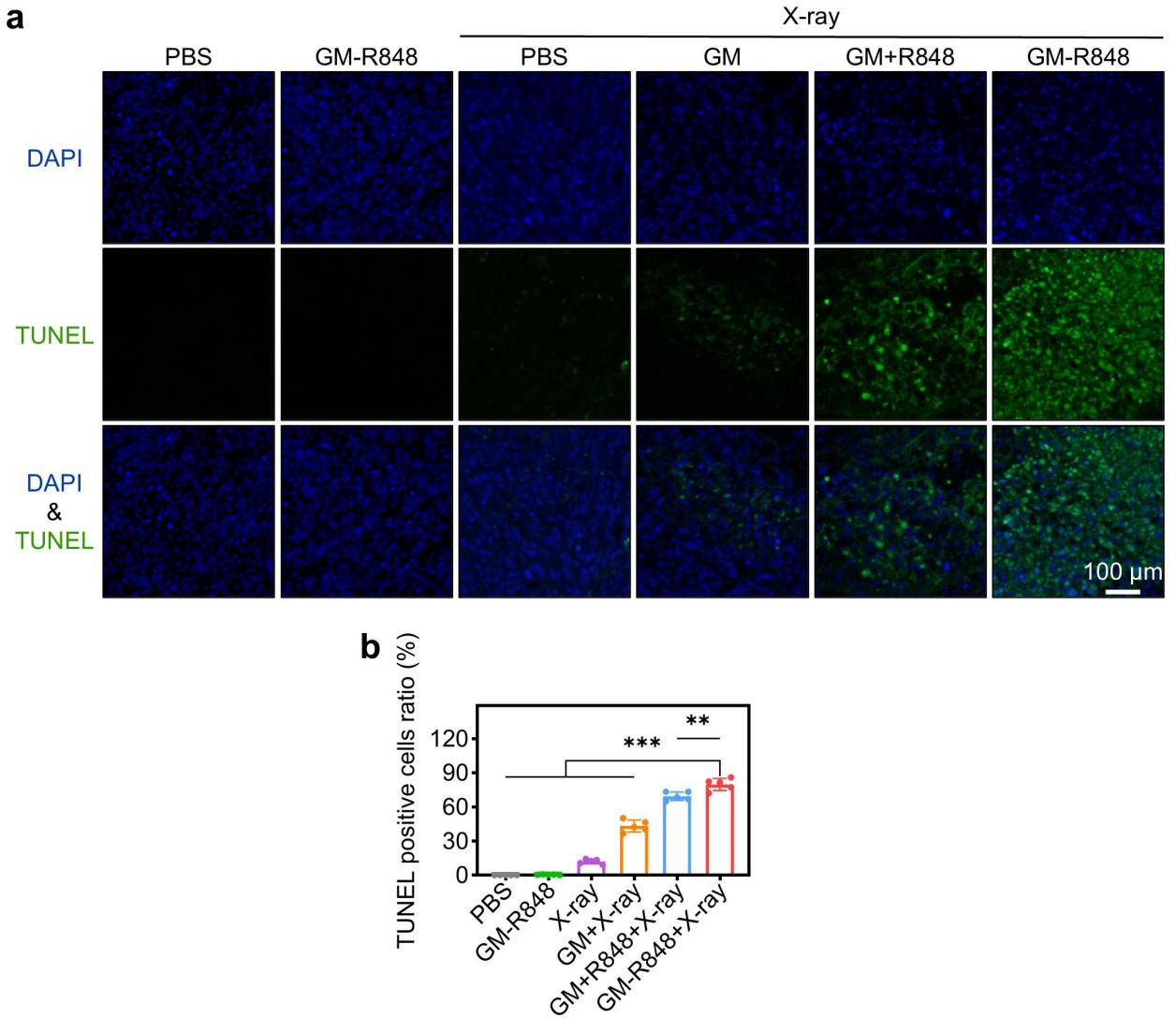


**Fig. S34.** (a) Representative TUNEL staining images for CT26 tumors in different treatment groups. (b) Quantification of the expression levels of TUNEL in different treatment groups (n = 5). ^**^*p* < 0.01, ^***^*p* < 0.001


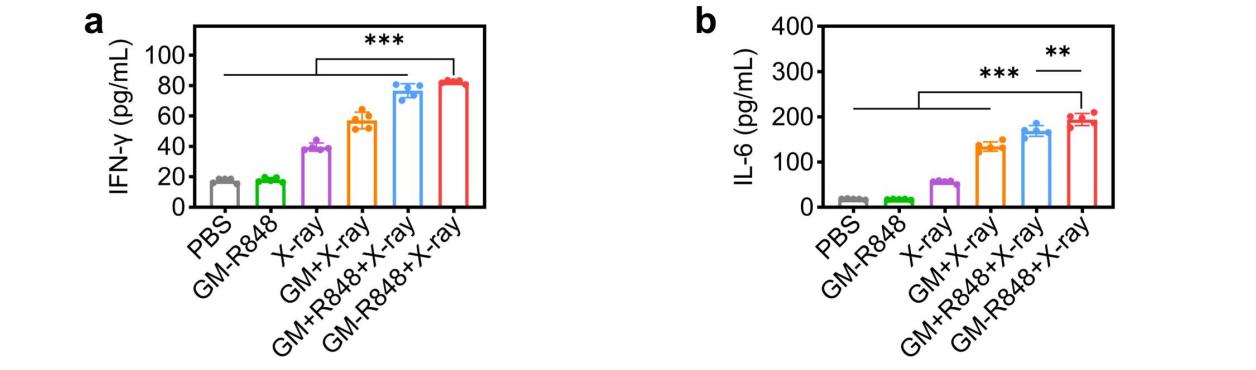


**Fig. S35.** The cytokine levels of (a) IFN-γ and (b) IL-6 in the ascites in the different treatment groups (n = 5). ^**^*p* < 0.01, ^***^*p* < 0.001


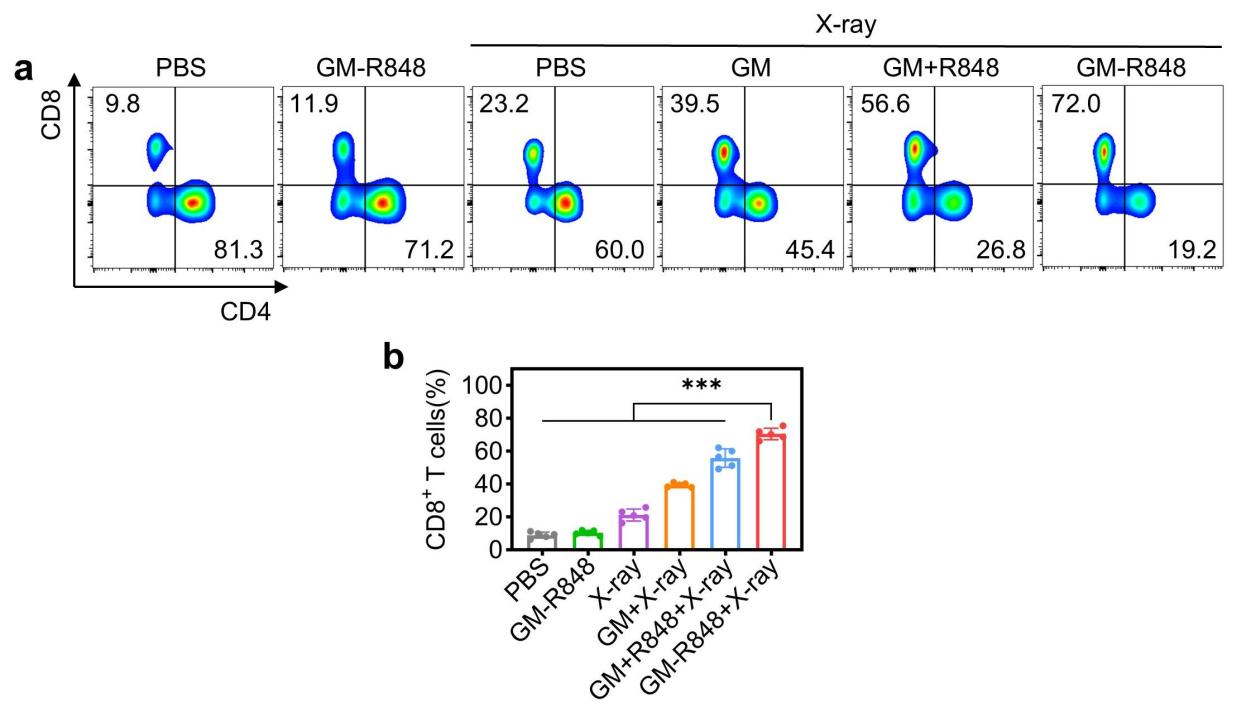


**Fig. S36.** Representative flow cytometry plots of (a) CD8^+^/CD4^+^ cells and the ratio of (b) CD8^+^ cells in the ascites for different treatment groups (n = 5). ^***^*p* < 0.001


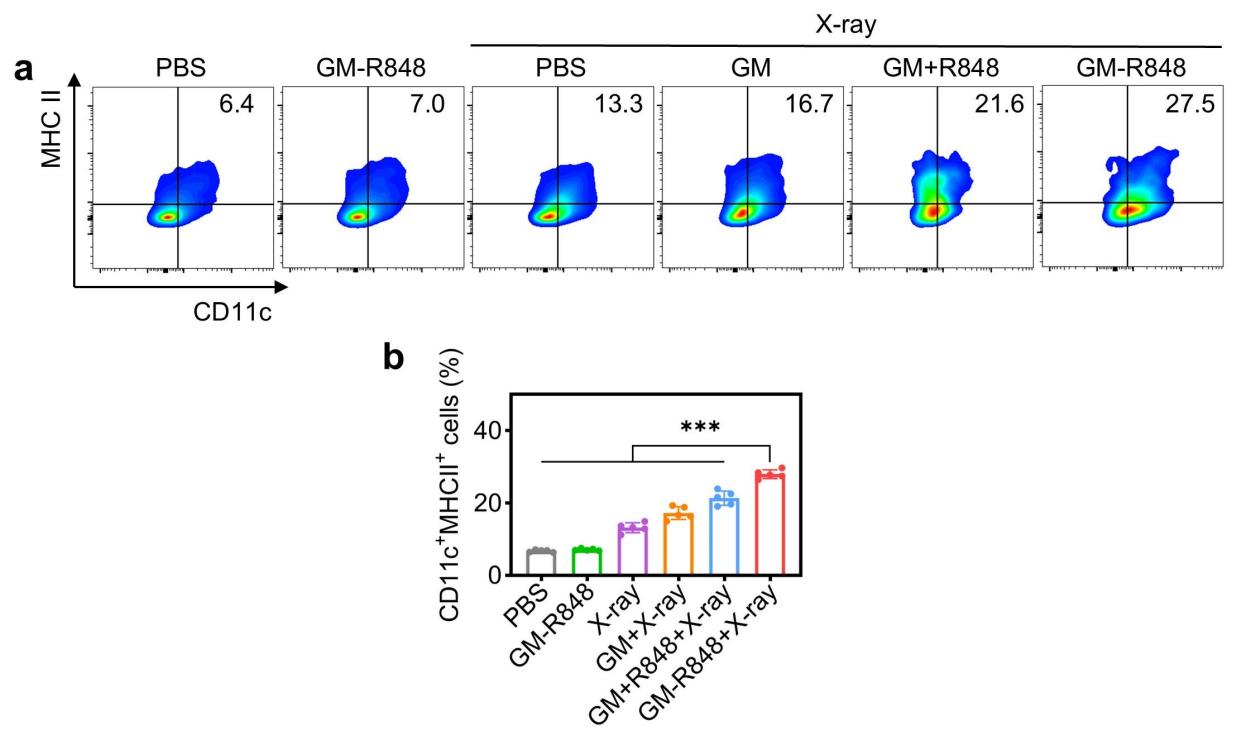


**Fig. S37.** (a) Representative flow cytometry plots of CD11c⁺MHCII^+^ cells and (b) the ratio of CD11c⁺MHCII⁺ cells in the ascites for different treatment groups (n = 5). ^***^*p* < 0.001


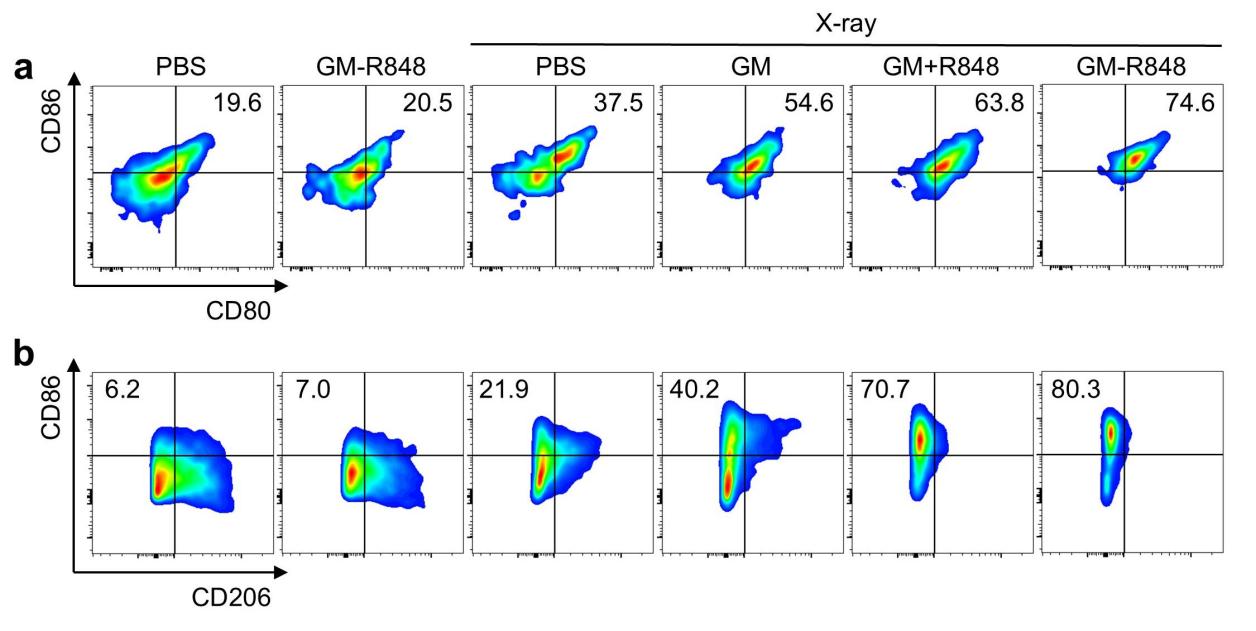


**Fig. S38.** Representative flow cytometry plots of (a) mature DCs and (b) M1-like macrophages in the ascites for different treatment groups (n = 5).


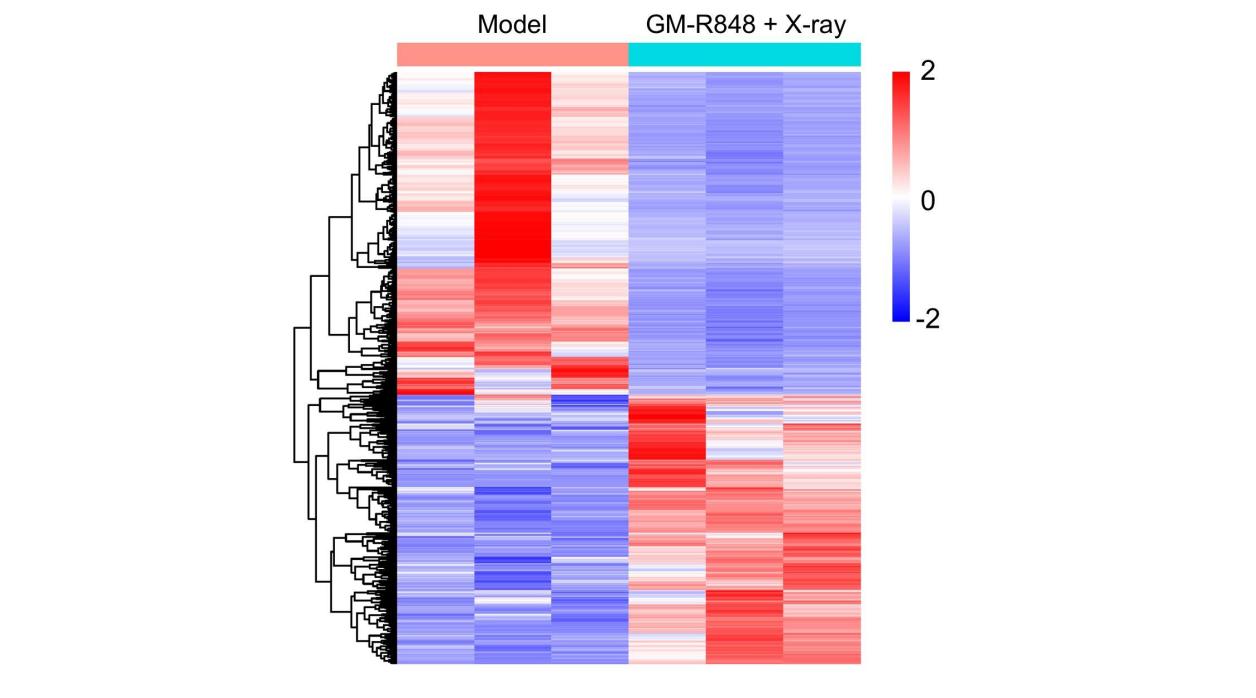


**Fig. S39.** The heatmap of differentially expressed genes between the Model group and the GM-R848 + X-ray group (n = 3).


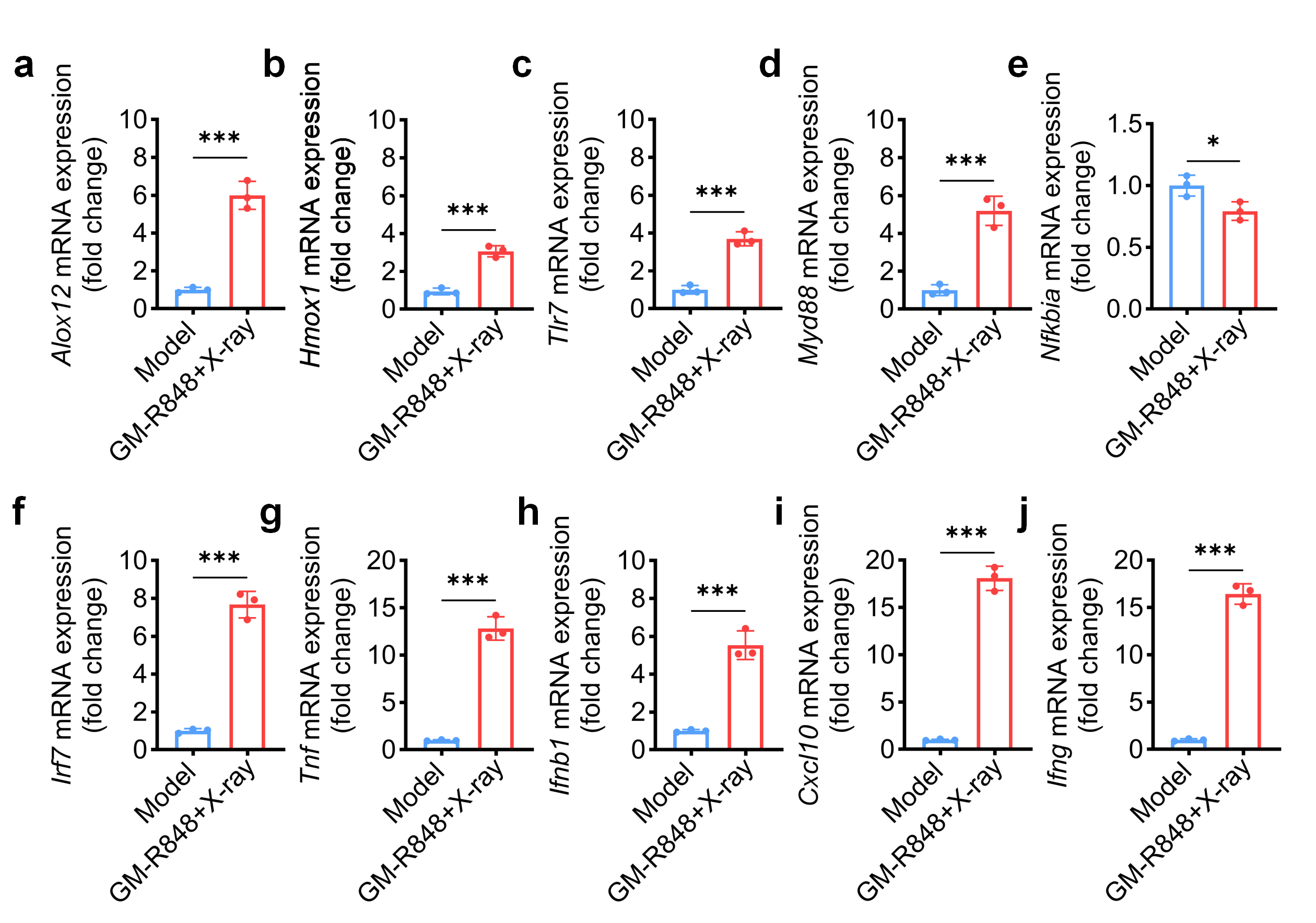


**Fig. S40.** (a-j) RT-qPCR analysis of mRNA levels between the Model group and the GM-R848 + X-ray group (n = 3). ^*^*p* < 0.05 ^***^*p* < 0.001

**Table S1. Partial primers used for RT-qPCR and PCR analysis.**

| Gene | Forward (F) / Reverse (R) | Sequence (5' to 3'') |
| --- | --- | --- |
| *β-actin* | F | CCTCTATGCCAACACAGTGC |
|  | R | ACATCTGCTGGAAGGTGGAC |
| *Alox12* | F | TTATGAAGCTCCGCTCTGGC |
|  | R | CCAAAGGCAAAGCACTCCAC |
| *Hmox1* | F | AAGCCGAGAATGCTGAGTTCA |
|  | R | GCCGTGTAGATATGGTACAAGGA |
| *Tlr7* | F | TCCACCCAACTTATCTTCAACGT |
|  | R | ACTCAGCGTCACCAATCTCC |
| *Myd88* | F | ATCGCTGTTCTTGAACCCTCG |
|  | R | CTCACGGTCTAACAAGGCCAG |
| *Nfkbia* | F | CTCCGAGACTTTCGAGGAAATAC |
|  | R | GCCATTGAAGTTGGTAGCCTTCA |
| *Irf7* | F | CCACGCTATACCATCTACCT |
|  | R | TATCCAGGGAAGACACACC |
| *Tnf* | F | CACGTCGTAGCAAACCACC |
|  | R | TGAGATCCATGCCGTTGGC |
| *Ifnb1* | F | CTTTGCTATTTTCAGACAAGATTCA |
|  | R | GCCAGGAGGTTCTCAACAAT |
| *Cxcl10* | F | CCAGAATCGAAGGCCATCAA |
|  | R | CATTTCCTTGCTAACTGCTTTCAG |
| *Ifng* | F | GGCATTTTGAAGAATTGGAAAG |
|  | R | TTTGGATGCTCTGGTCATCTT |
